# Enzastaurin inhibits ABCB1-mediated drug efflux independently of effects on protein kinase C signalling and the cellular p53 status

**DOI:** 10.18632/oncotarget.2889

**Published:** 2015-02-13

**Authors:** Martin Michaelis, Florian Rothweiler, Nadine Löschmann, Mohsen Sharifi, Taravat Ghafourian, Jindrich Cinatl

**Affiliations:** ^1^ Institut für Medizinische Virologie, Klinikum der Goethe-Universität, Frankfurt am Main, Germany; ^2^ Centre for Molecular Processing and School of Biosciences, University of Kent, Canterbury, UK; ^3^ Medway School of Pharmacy, Universities of Kent and Greenwich, Chatham Maritime, Kent, UK

**Keywords:** ONC201, cancer drug, TIC10, NSC350625

## Abstract

The PKCβ inhibitor enzastaurin was tested in parental neuroblastoma and rhabdomyosarcoma cell lines, their vincristine-resistant sub-lines, primary neuroblastoma cells, ABCB1-transduced, ABCG2-transduced, and p53-depleted cells. Enzastaurin IC_50_s ranged from 3.3 to 9.5 μM in cell lines and primary cells independently of the ABCB1, ABCG2, or p53 status. Enzastaurin 0.3125 μM interfered with ABCB1-mediated drug transport. PKCα and PKCβ may phosphorylate and activate ABCB1 under the control of p53. However, enzastaurin exerted similar effects on ABCB1 in the presence or absence of functional p53. Also, enzastaurin inhibited PKC signalling only in concentrations ≥ 1.25 μM. The investigated cell lines did not express PKCβ. PKCα depletion reduced PKC signalling but did not affect ABCB1 activity. Intracellular levels of the fluorescent ABCB1 substrate rhodamine 123 rapidly decreased after wash-out of extracellular enzastaurin, and enzastaurin induced ABCB1 ATPase activity resembling the ABCB1 substrate verapamil. Computational docking experiments detected a direct interaction of enzastaurin and ABCB1. These data suggest that enzastaurin directly interferes with ABCB1 function. Enzastaurin further inhibited ABCG2-mediated drug transport but by a different mechanism since it reduced ABCG2 ATPase activity. These findings are important for the further development of therapies combining enzastaurin with ABC transporter substrates.

## INTRODUCTION

Enzastaurin (also known as LY317615) was synthesised as protein kinase C (PKC) β inhibitor based on the structure of staurosporine, a natural compound known to interfere with PKC signalling [1]. In pre-clinical models, enzastaurin displayed activity against cancer cells from different entities including carcinomas, glioblastoma, melanoma, and different haematological malignancies as well as anti-angiogenic effects [2–13]. The substance has been investigated for its effects against various cancer types in clinical trials [4, 13–22].

We had previously shown that enzastaurin activates glycogen synthase kinase (GSK) 3β in natural killer cells and in turn reduces their activity [23]. To test the effects of (potential) anti-cancer agents in the context of cellular chemoresistance mechanisms, we have established the Resistant Cancer Cell Line (RCCL) collection, a collection of cell lines from 15 different cancer entities with acquired resistance to various cytotoxic and targeted anti-cancer drugs (http://www.kent.ac.uk/stms/cmp/RCCL/RCCLabout.html) including cell lines derived from the paediatric cancer entities neuroblastoma and rhabdomyosarcoma. Significant subgroups of patients suffering from these cancers are high-risk patients with very poor prognosis [24–27]. Vincristine is a constituent of therapy regimens for both neuroblastoma and rhabdomyosarcoma [24–28]. Here, we tested enzastaurin alone or in combination with vincristine in a panel of parental neuroblastoma and rhabdomyosarcoma cell lines and their vincristine-resistant sub-lines.

## RESULTS

### Influence of enzastaurin on viability of neuroblastoma and rhabdomyosarcoma cells

Enzastaurin interfered with the viability of chemosensitive and chemoresistant neuroblastoma and rhabdomyosarcoma cells in similar concentrations, enzastaurin IC_50_ values ranged from 3.74 to 8.20 μM (Table 1). Similar results were obtained in primary MYCN-amplified neuroblastoma cells (Table 2).

**Table 1:** Influence of enzastaurin on neuroblastoma and rhabdomyosarcoma cell viability Expressed as concentration that reduces cell viability (determined by MTT assay) after a 5 day incubation period by 50% (IC_50_)

Cell line	p53 status	ABCB1	IC_50_^(1)^ enzastaurin (μM)
UKF-NB-3	wild-type	− ^(2)^	6.31 ± 0.98^(3)^
UKF-NB-3^r^VCR^10^	mut (C135F)^(4)^	+	5.33 ± 1.09
UKF-NB-3^ABCB1 (5)^	wild-type	+	9.46 ± 1.89
UKF-NB-3^Cer2 (6)^	wild-type	−	8.44 ± 0.95
UKF-NB-3^ABCG2 (7)^	wild-type	−	5.61 ± 1.03
UKF-NB-3^iG2 (8)^	wild-type	−	5.31 ± 0.88
UKF-NB-3^p53shRNA (9)^	depleted	−	3.53 ± 0.69
UKF-NB-3^scrshRNA (10)^	wild-type	−	4.71 ± 0.73
UKF-NB-2	wild-type	−	3.74 ± 0.48
UKF-NB-2^r^VCR^10^	wild-type	+	6.67 ± 1.25
KFR	wild-type	−	5.04 ± 0.84
KFR^r^VCR^10^	wild-type	+	4.52 ± 0.71
Rh30	mut (R273C)	−	6.84 ± 0.96
Rh30^r^VCR^10^	mut (R273C)	+	8.20 ± 1.07

(1) concentration that reduces cell viability (determined by MTT assay) after a 5 day incubation period by 50%

(2) ABCB1 expression levels are presented in Suppl. Table 6

(3) Values are mean ± S.D.

(4) mut=mutated, kind of mutation in brackets

(5) UKF-NB-3 cells transduced with a lentiviral vector encoding for the *ABCB1* gene

(6) UKF-NB-3 cells transduced with an empty lentiviral control vector, serving as transduction control for UKF-NB-3^ABCB1^

(7) UKF-NB-3 cells transduced with a lentiviral vector encoding for the *ABCG2* gene

(8) UKF-NB-3 cells transduced with an empty lentiviral control vector, serving as transduction control for UKF-NB-3^ABCG2^

(9) UKF-NB-3 cells transduced with a lentiviral vector encoding for shRNA directed against p53

(10) UKF-NB-3 cells transduced with a lentiviral vector encoding scrambled (non-targeted) shRNA.

**Table 2:** Enzastaurin concentrations that reduce viability of primary MYCN-amplified neuroblastoma cells by 50% (IC_50_)

	IC_50_ (μM)
isolate 1	6.2 ± 2.3
isolate 2	4.4 ± 1.3
isolate 3	3.3 ± 1.4
isolate 4	7.6 ± 2.9

ABCB1 and ABCG2 are major ATP-binding cassette (ABC) transporters that are involved in the passage of drugs, xenobiotics, and food constituents through cellular and tissue barriers and consequently in their absorption, distribution, and excretion. Moreover, ABCB1 and ABCG2 are frequently found highly expressed on cancer cells playing an important role in cancer cell chemoresistance [29–31]. p53 is a major tumour suppressor gene. Loss-of-p53 function has been associated with decreased drug sensitivity in cancers including neuroblastoma [32, 33]. However, neither p53 functionality nor ABCB1 or ABCG2 expression status significantly modified enzastaurin sensitivity of the investigated cell lines (Table 1).

### Enzastaurin sensitises ABCB1-expressing cells to cytotoxic ABCB1 substrates

**Figure 1:**
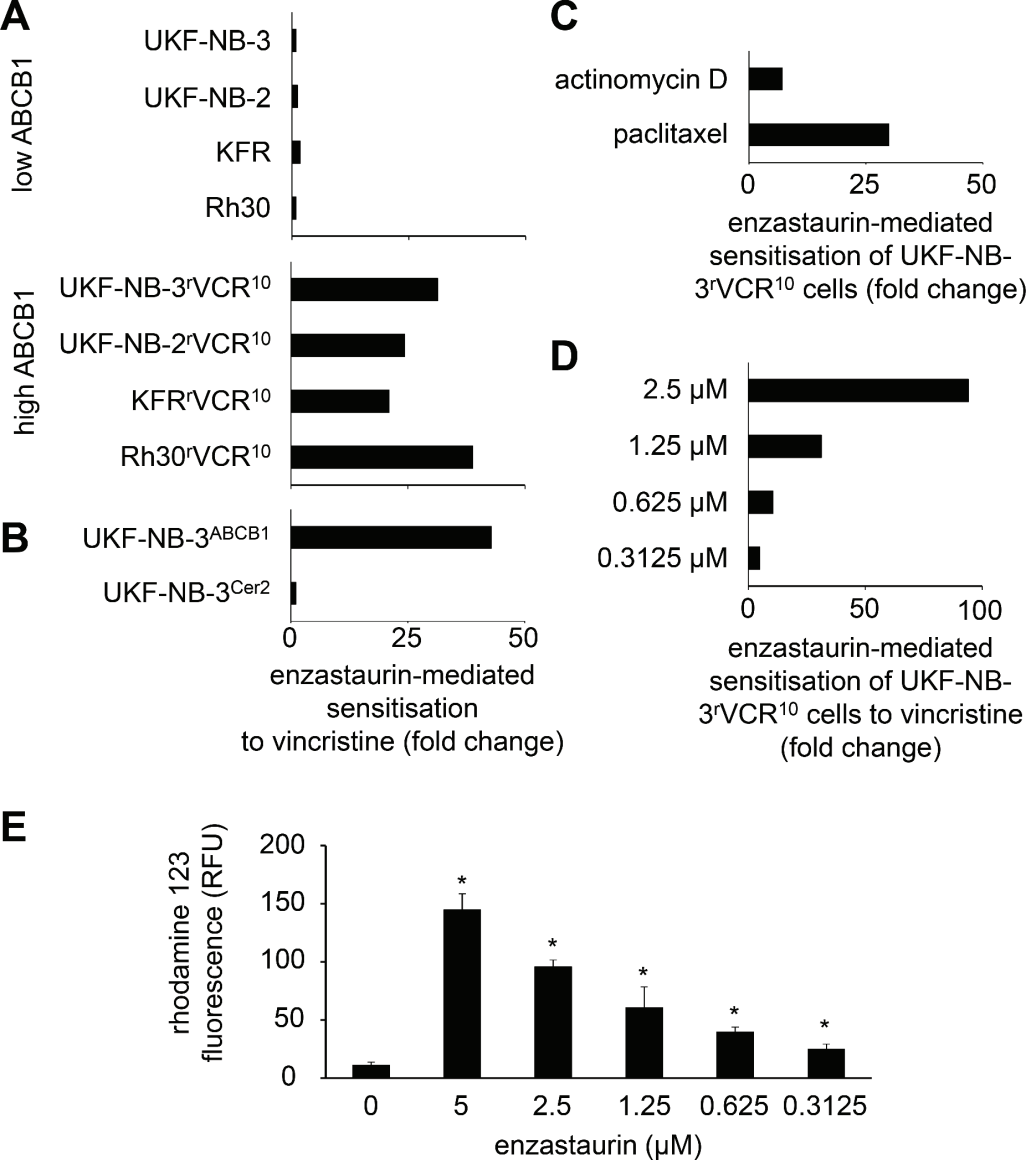
Influence of enzastaurin on drug sensitivity in ABCB1-expressing cells **(A)** Sensitisation of low and high ABCB1 expressing cells to the ABCB1 substrate vincristine by enzastaurin 1.25 μM, a concentration that did not influence viability of the investigated cell lines (fold change IC_50_ vincristine/IC_50_ vincristine in the presence of enzastaurin); **(B)** sensitisation of UKF-NB-3 cells transduced with a lentiviral vector encoding for *ABCB1* (UKF-NB-3^ABCB1^) or an empty control vector (UKF-NB-3^Cer2^) to vincristine by enzastaurin 1.25 μM (fold change IC_50_ vincristine/IC_50_ vincristine in the presence of enzastaurin). Numerical data for A) and B) are presented in Suppl. Table 1. **(C)** Sensitisation of UKF-NB-3^r^VCR^10^ cells to the cytotoxic ABCB1 substrates actinomycin D and paclitaxel by enzastaurin 1.25 μM (fold change IC_50_ drug alone/IC_50_ drug in the presence of enzastaurin). Numerical data are presented in Suppl. Table 2. **(D)** Sensitisation of UKF-NB-3^r^VCR^10^ cells to vincristine by enzastaurin (fold change IC_50_ vincristine /IC_50_ vincristine in the presence of enzastaurin). Numerical data are presented in Suppl. Table 3. **(E)** Influence of enzastaurin on accumulation of rhodamine 123 (0.5 μM; a fluorescent ABCB1 substrate) in ABCB1-expressing UKF-NB-3^r^VCR^10^ cells as detected by flow cytometry (RFU = relative fluorescence units). *P < 0.05 relative to rhodamine alone.

Next, the influence of enzastaurin was tested on the vincristine sensitivity of neuroblastoma and rhabdomyosarcoma cells. Enzastaurin 1.25 μM, a concentration that did not significantly affect cell line viability, sensitised ABCB1-expressing vincristine-resistant cells to the ABCB1 substrate vincristine but did not substantially change the vincristine sensitivity of the respective parental cell lines that express low levels of ABCB1 (Figure 1A, Suppl. Table 1). Effects were similar in vincristine-adapted, ABCB1-expressing cells and in UKF-NB-3^ABCB1^ cells transduced with a lentiviral vector encoding for *ABCB1* (Figure 1B, Suppl. Table 1).

ABCB1 recognises a broad range of structurally different substrates. In concordance, enzastaurin 0.625 μM and 1.25 μM also dose-dependently sensitised ABCB1-expressing UKF-NB-3^r^VCR^10^ cells (that do not express ABCC1, ABCC2, ABCC3, ABCC5, or ABCG2, data not shown) to the alternative cytotoxic ABCB1 substrates paclitaxel and actinomycin D (Figure 1C, Suppl. Table 2). Enzastaurin further sensitised ABCB1-expressing UKF-NB-3^r^VCR^10^, UKF-NB-2^r^VCR^10^, KFR^r^VCR^10^, and Rh30^r^VCR^10^ cells (but not non-ABCB1-expressing UKF-NB-3, UKF-NB-2, KFR, and Rh30 cells) to vincristine in a dose-dependent manner. Enzastaurin concentrations as low as 0.3125 μM were found to enhance vincristine activity (Figure 1D, Suppl. Table 3A–3D).

Finally, we investigated the influence of enzastaurin on the efflux of the fluorescent ABCB1 substrate rhodamine 123 in ABCB1-expressing UKF-NB-3^r^VCR^10^ cells. Enzastaurin caused a concentration-dependent increase in rhodamine 123 fluorescence in the UKF-NB-3^r^VCR^10^ cells (Figure 1E) but did not affect ABCB1 expression (data not shown).

### Direct interaction of enzastaurin with ABCB1

**Figure 2:**
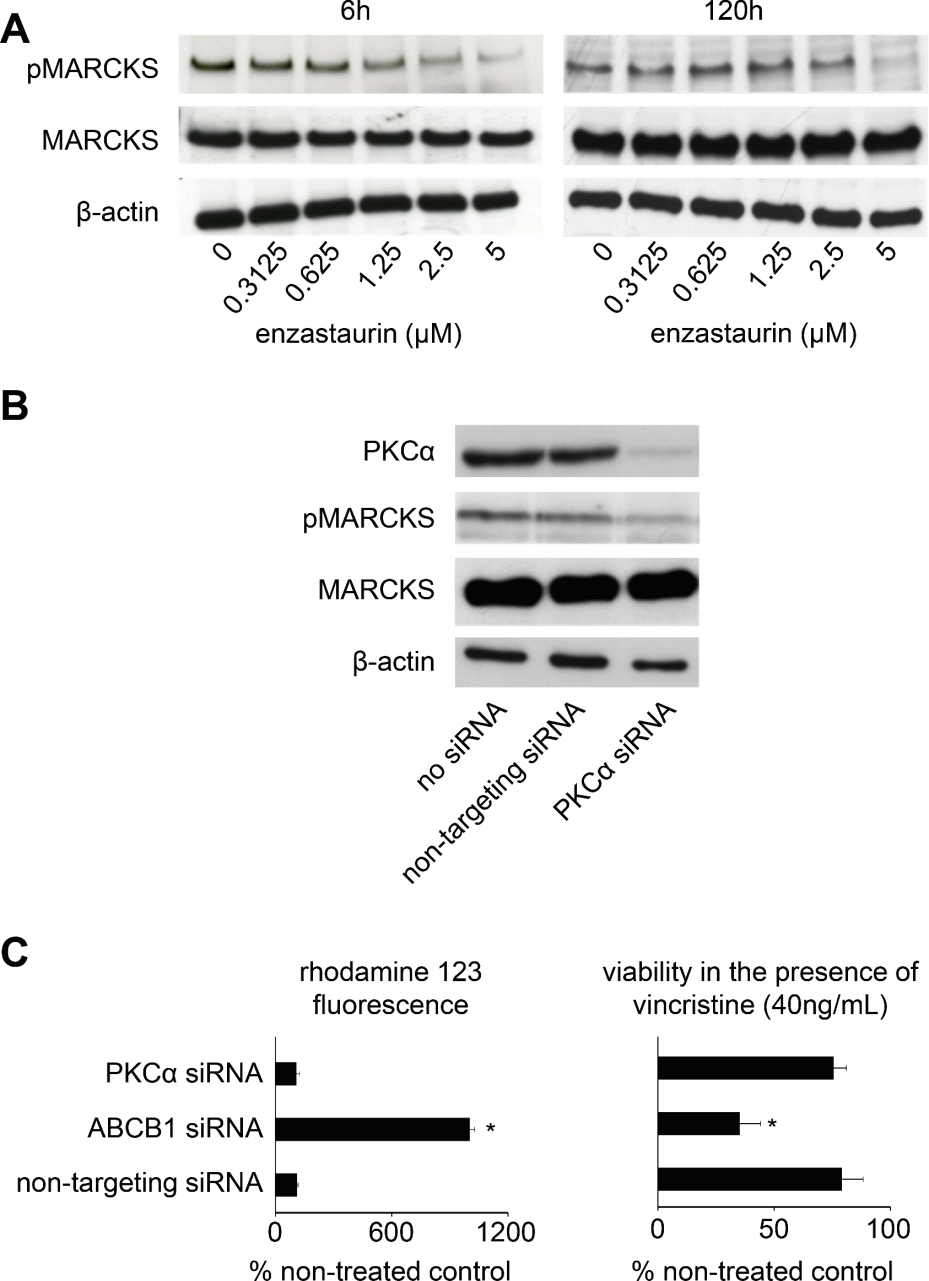
Investigation of PKC signalling **(A)** Enzastaurin inhibits PKC activity in concentrations ≥ 1.25 μM in UKF-NB-3^r^VCR^10^ cells after enzastaurin treatment for 6 h and in a concentration of 5 μM after 120 h. Western blots indicate protein levels of the PKC target MARCKS, its phosphorylated form (pMARCKS), and β-actin (loading control). Effects on ABCB1 function were observed at an enzastaurin concentration of 0.3125 μM (Figure 1D, Suppl. Table 3). **(B)** Effects of siRNA-mediated PKCα depletion on MARCKS phosphorylation determined in UKF-NB-3^r^VCR^10^ cells 48 h after transfection; **(C)** siRNA directed against ABCB1 (but not siRNA directed against PKCα) increases (1) accumulation of the fluorescent ABCB1 substrate rhodamine 123 (0.5 μM) in ABCB1-expressing UKF-NB-3^r^VCR^10^ cells and (2) the sensitivity of UKF-NB-3^r^VCR^10^ cells to the cytotoxic ABCB1 substrate vincristine. Effects of siRNAs on ABCB1 expression are presented in Suppl. Figure 1. (*P < 0.05) relative to non-targeting siRNA.

Previous reports had indicated that PKCα or PKCβ may promote ABCB1 function by phosphorylation [34, 35]. Therefore, enzastaurin may affect ABCB1 function through direct interaction with ABCB1 and/or inhibition of PKC-mediated ABCB1 phosphorylation. Enzastaurin affected ABCB1 function in concentrations as low as 0.3125 μM (Figure 1D, Figure 1E, Suppl. Table 3). Since enzastaurin was shown to inhibit PKCβ enzyme activity with an IC_50_ of 0.03 μM and PKCα activity with an IC_50_ of 0.8 μM in isolated enzyme assays [1], enzastaurin-mediated effects on PKCα signalling are unlikely to be responsible for the reduced ABCB1 activity. Myristoylated alanine-rich C-kinase substrate (MARCKS) is a PKC substrate, and MARCKS phosphorylation is a surrogate parameter for PKC activity [3, 4]. Enzastaurin inhibited MARCKS phosphorylation in UKF-NB-3^r^VCR^10^ cells only in concentrations of 1.25 μM or higher after 6 h of incubation. After 120 h, only an enzastaurin concentration of 5 μM reduced MARKS phosphorylation (Figure 2A). Since enzastaurin inhibits ABCB1 function in concentrations as low as 0.3125 μM (Figure 1D, Suppl. Table 3), this finding suggests that the enzastaurin-mediated inhibition of ABCB1 function may not be the consequence of inhibition of PKC-mediated ABCB1 phosphorylation.

Next, we wanted to test whether interference with PKC signalling is sufficient to interfere with ABCB1 function in our model. Although we readily detected PKCβ in K562 cells that had served as positive control, we were not able to detect PKCβ in UKF-NB-3^r^VCR^10^ cells (Suppl. Figure 1A). PKCα was present, and siRNA-mediated PKCα depletion inhibited PKC signalling as indicated by decreased levels of phosphorylated MARCKS (Figure 2B, Suppl. Figure 1B). However, siRNA-mediated depletion of PKCα did (in contrast to ABCB1 depletion) not increase rhodamine 123 accumulation in or vincristine sensitivity of ABCB1-expressing UKF-NB-3rVCR10 cells (Figure 2C, Suppl. Figure 1C). Similar results were obtained in UKF-NB-3^ABCB1^ cells (Suppl. Figure 2A–2C).

**Figure 3:**
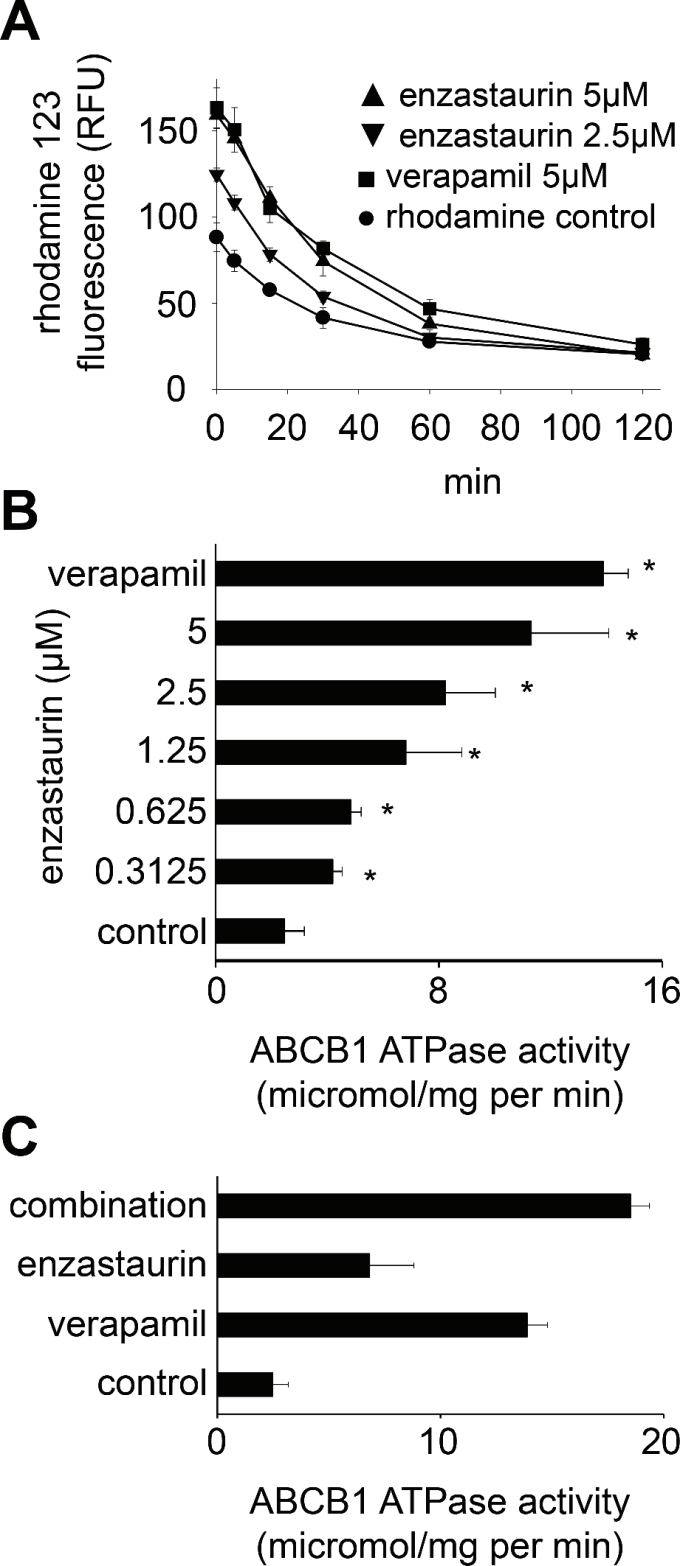
Direct interaction of enzastaurin with ABCB1 **(A)** Time kinetics of rhodamine 123 (0.5 μM) fluorescence in UKF-NB-3^r^VCR^10^ cells after incubation for 60 min with rhodamine 123 alone (rhodamine control), enzastaurin 5 μM or 2.5 μM plus rhodamine 123, or verapamil 5 μM (known ABCB1 substrate serving as control) plus rhodamine 123 after wash-out as detected by flow cytometry (RFU = relative fluorescence units). *P < 0.05 relative to rhodamine alone; **(B)** ABCB1 ATPase activity in isolated membranes in the presence of verapamil 5 μM or enzastaurin. *P < 0.05 relative to non-treated control; **(C)** ABCB1 ATPase activity in non-treated isolated membranes or in the presence of verapamil 5 μM, enzastaurin 1.25 μM, or the combination of verapamil 5 μM and enzastaurin 1.25 μM. *P < 0.05 relative to single treatment with verapamil and enzastaurin.

After incubation of UKF-NB-3^r^VCR^10^ cells with rhodamine 123 and enzastaurin, the wash-out of extracellular enzastaurin (and rhodamin 123) resulted in a rapid decrease of cellular rhodamine 123 fluorescence in a similar fashion like the wash out of the known ABCB1 substrate verapamil (Figure 3A). In addition, enzastaurin 0.3125 μM significantly increased ABCB1 ATPase activity (Figure 3B). The combination of enzastaurin and verapamil further enhanced ABCB1 ATPase activity (Figure 3C). Taken together, these data suggest that enzastaurin interferes with ABCB1 predominantly through direct interaction with ABCB1, possibly being an ABCB1 substrate.

### Docking experiments suggest a direct interaction of enzastaurin with ABCB1

Enzastaurin was docked into the homology model of human ABCB1 and the three x-ray structures of mouse Abcb1a, with the binding sites defined using the positions of the co-crystallised ligands, QZ59-RRR and QZ59-SSS, and the verapamil binding site (residues protected from methanethiosulfonate (MTS) labelling by verapamil) as described by Aller et al. [36]. Results indicate a strong interaction between enzastaurin and ABCB1. The docking scores for the top five docking poses ranged between −16.60 and −9.01 kcal/mol for mouse, and between −13.30 and −10.42 kcal/mol for human ABCB1 (Suppl. Table 4). Docking enzastaurin into 3G60 and 3G61 generated the lowest binding scores. The protein structures 3G61 and 3G60 are the structures co-crystallised with QZ59-SSS and QZ59-RRR respectively, and have a x-ray resolutions of 4.35 Å and 4.40 Å. 3G5U is the apo-structure with a higher x-ray resolution of 3.80 Å. Suppl. Table 4 also shows that the best binding scores in all cases are achieved when the biding site is defined using the co-crystallised QZ59-SSS (see the average of the top five poses in Suppl. Table 4). The only exception to this is the top pose obtained for 3G60 when docked into QZ59-RRR binding site. The energy of the top pose can be as low as −16.60 when the binding site residues of QZ59-RRR have been selected to define the binding pocket for enzastaurin (Suppl. Table 4).

**Figure 4:**
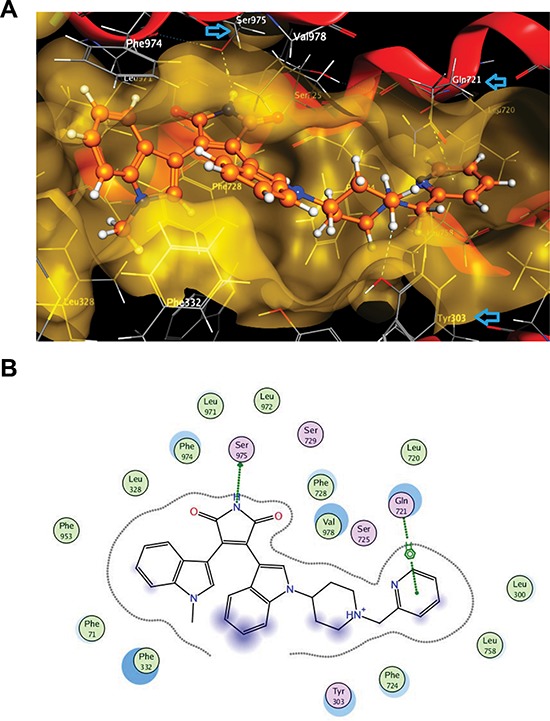
Interaction of enzastaurin with ABCB1 as indicated by docking experiments **(A)** 3D view of a portion of the active site of mouse abc1b1 (3G60) at the lowest docking energy with docked enzastaurin in the binding pocket; blue arrows in this pose indicate residues (Ser925, Tyr303 and Gln721) that have strong interactions with enzastaurin (limited within 4.5 Å); **(B)** 2D ligand interaction diagram for enzastaurin with mouse Abcb1a using default settings of MOE software. The polar and non-polar residues are indicated by pink or green coloured amino acids respectively. Hydrogen bonding is indicated by green dotted arrow, while arene-H interaction is shown by green dotted line. The proximity contour is the dotted line surrounding the ligand. Blue shadows in of the residues indicate the receptor exposure differences by the size and intensity of the quoits discs. The directions of the shadow indicate the directions of the amino acids towards the ligands. The blue clouds around the ligand atoms indicate that are exposed to the solvent.

Suppl. Table 5 shows the ligand interaction report indicating specific interactions between structural elements of the enzastaurin structure and ABCB1 residues in the top docking poses. A summary of the residues involved in the enzastaurin interaction with mouse Abcb1a has been plotted in Figure 4A. The figure indicates that the most prevalent interacting residues in mouse Abcb1a are Phe71, Phe728, and Phe974. Notably, Phe728 and Phe974 are the amino acids reported by Aller et al. [36] to be involved in the QZ59-RRR binding site. These three Phe residues are mostly involved in π-π interactions, with fewer occurrences of π-H or π-cation interactions with the ligand (Suppl. Table 5). An example of a top scoring docking pose for the interaction of enzastaurin with mouse Abcb1a is shown in Figure 4B. The Phe residues 71, 728 and 974 are present in the binding site with the shadows indicating that in the absence of the ligand these amino acids are highly exposed to the solvent, but the presence of the ligand greatly reduces the solvent accessible surface area (Figure 4B). In addition, there are several other lipophilic residues in the close proximity of the ligand with receptor exposure shadows indicating the possibility of several hydrophobic interaction points (Figure 4B). The pyridine ring in the ligand has a strong arene-H interaction with Gln721, while the pyrrole ring is engaged in H-bonding interaction with Ser975 (Figure 4).

### Effects of enzastaurin on ABCG2

**Figure 5:**
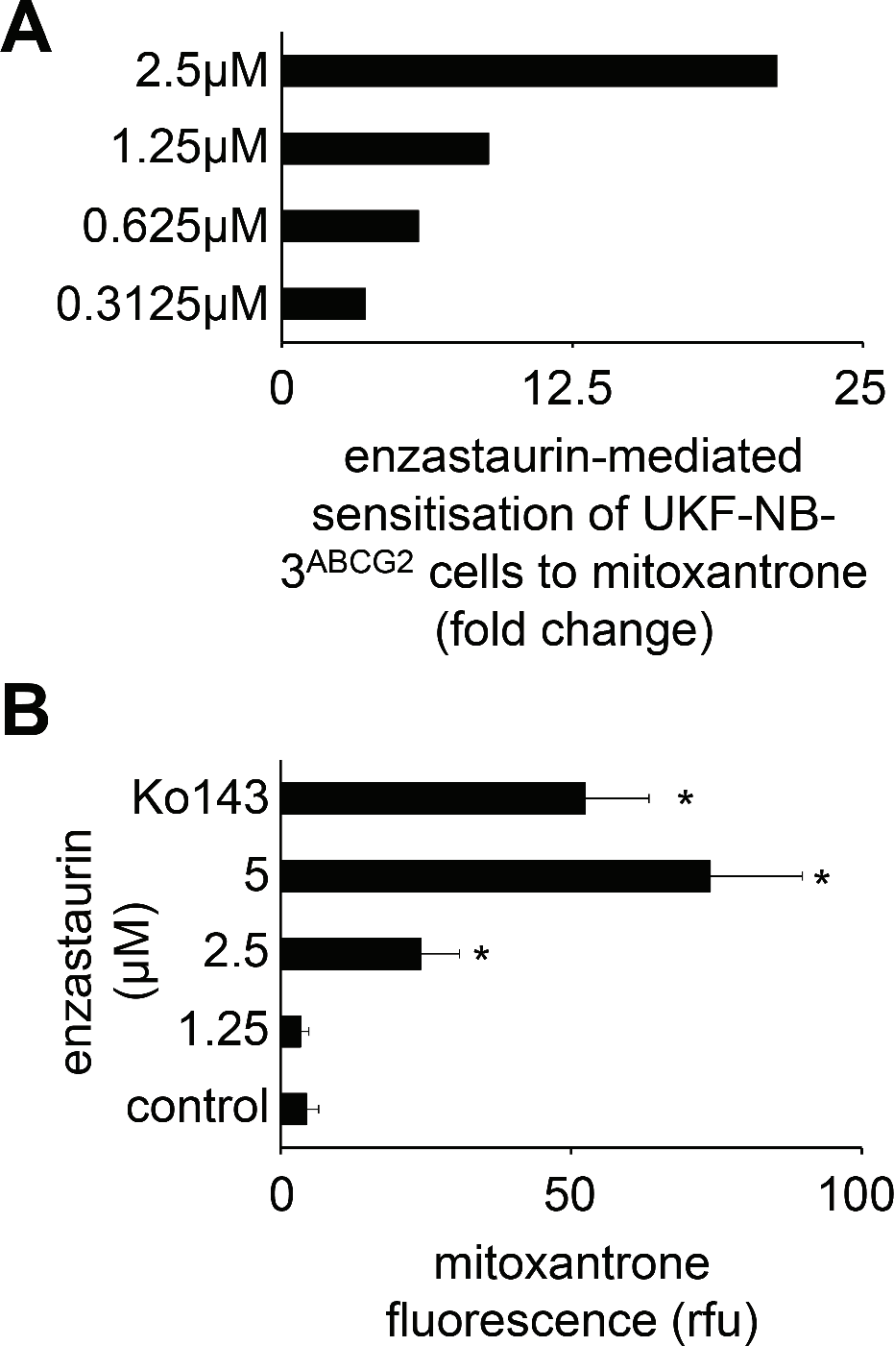
Influence of enzastaurin on ABCG2 function **(A)** Sensitisation of UKF-NB-3 cells transduced with a lentiviral vector encoding for ABCG2 (UKF-NB-3^ABCG2^) to the ABCG2 substrate mitoxantrone by enzastaurin concentrations that did not influence UKF-NB-3^ABCG2^ cell viability (fold change IC_50_ mitoxantrone/IC_50_ mitoxantrone in the presence of enzastaurin), numerical data are presented in Suppl. Table 6; **(B)** Influence of enzastaurin on accumulation of mitoxantrone (40 μM; a fluorescent ABCG2 substrate) in UKF-NB-3^ABCG2^ cells as detected by flow cytometry (RFU = relative fluorescence units). The ABCG2 inhibitor Ko143 (1 μM) served as positive control. *P < 0.05 relative to mitoxantrone alone.

**Figure 6:**
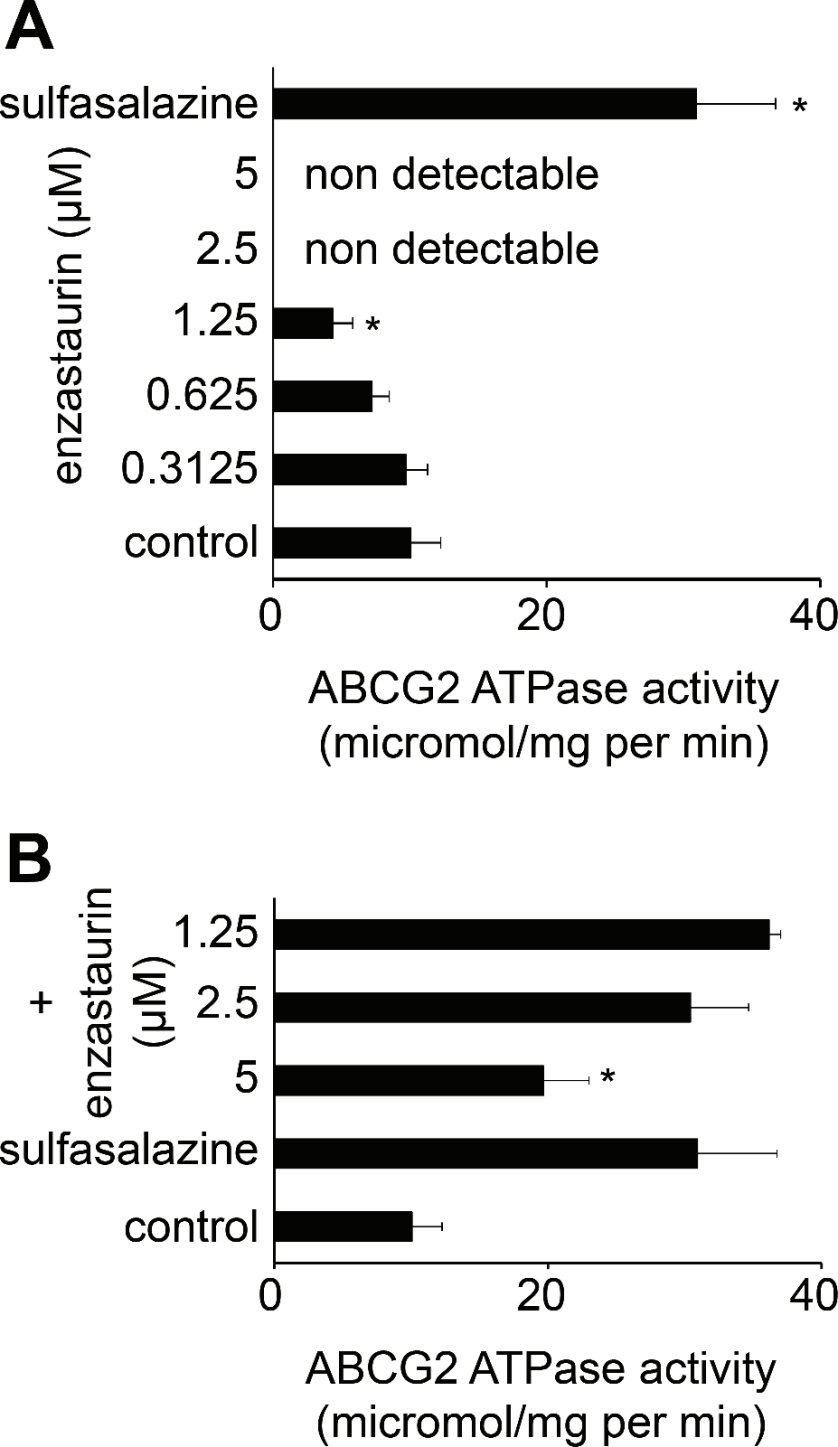
Effects of enzastaurin on the ABCG2 ATPase **(A)** ABCG2 ATPase activity in isolated membranes in the presence of enzastaurin or the ABCG2 substrate sulfasalazine 10 μM. *P < 0.05 relative to non-treated control; **(B)** ABCG2 ATPase activity in non-treated isolated membranes or in the presence of sulfasalazine 10 μM alone or in combination with enzastaurin. *P < 0.05 relative to single treatment with sulfasalazine.

Finally, we showed that enzastaurin also concentration-dependently sensitised ABCG2-transduced UKF-NB-3 (UKF-NB-3^ABCG2^) cells (but not UKF-NB-3 or control vector-transduced UKF-NB-3^iG2^ cells) to toxicity induced by the cytotoxic ABCG2 substrate mitoxantrone (Figure 5A, Suppl. Table 6A–6C). In addition, enzastaurin enhanced mitoxantrone accumulation in UKF-NB-3^ABCG2^ cells but not in UKF-NB-3^iG2^ cells as determined by flow cytometry (Figure 5B; Suppl. Figure 3). While enzastaurin had enhanced ABCB1 ATPase activity, it inhibited basal and sulfasalazine-induced ABCG2 activity (Figure 6A and 6B) indicating that enzastaurin inhibits ABCB1 and ABCG2 activity through different mechanisms.

## DISCUSSION

PKC signalling was reported to be relevant in neuroblastoma and rhabdomyosarcoma [37–40]. In this report, IC_50_ values between 3.7 and 8.2 μM were determined for the PKCβ inhibitor enzastaurin in a panel of parental neuroblastoma and rhabdhomyosarcoma cell lines and their vincristine-resistant sub-lines and IC_50_s between 3.3 and 9.5 μM in primary neuroblastoma cells. These concentrations are in the range of those reported to be effective in other cancer entities [2, 6]. Notably, enzastaurin activity was not affected by ABC transporters ABCB1 or ABCG2 or the cellular p53 status. With regard to p53, our results are in line with a recent study that showed that the effects of enzastaurin monotherapy did not differ between HCT116^p53wt^ and HCT^p53−/−^ cells [41]. However, since enzastaurin plasma levels of 1 − 2 μM were reported to be achievable in patients [14, 42], enzastaurin may rather not be a candidate for single therapy in neuroblastoma or rhabdomyosarcoma.

Enzastaurin concentrations as low as 0.3125 μM sensitised ABCB1-expressing cells (but not non-ABCB1-expressing cells) to toxicity induced by the ABCB1 substrate vincristine. Enzastaurin also sensitised ABCB1-expressing cells to the structurally differing cytotoxic ABCB1 substrates paclitaxel and actinomycin D. Notably, enzastaurin exerted more pronounced effects on vincristine- and paclitaxel-mediated toxicity than on actinomycin D-induced toxicity. The exact molecular mechanisms underlying these differences are not clear. It is known that the mode and/or strength of ABCB1 interaction may differ among ABCB1 substrates and ABCB1 modulators. Certain ABCB1 modulators were shown to exert differing effects on the cellular accumulation of distinct ABCB1 substrates [43–45].

Moreover, enzastaurin caused a dose-dependent accumulation of the fluorescent ABCB1 substrate rhodamine 123 in ABCB1-expressing cells. Again, significant effects were determined at an enzastaurin concentration of 0.3125 μM. These data indicate that enzastaurin interferes with ABCB1-mediated drug transport. This is of potential clinical relevance. ABCB1 is expressed at virtually every tissue and organ barrier and influences the absorption, distribution, and excretion of drugs, xenobiotics, and food constituents [29, 31]. Therefore, enzastaurin may affect the pharmacokinetics of co-administered drugs including anti-cancer drugs and drugs non-related to cancer. Moreover, ABCB1 is frequently found highly expressed on cancer cells playing an important role in cancer cell drug resistance [30]. Seemingly, our data are in accordance with unpublished internal data from Eli Lilly [46].

Previous reports had shown that PKCα and PKCβ may promote ABCB1 function by phosphorylation [34, 35]. However, other data did not support this [47, 48], and further reports even suggested that PKC signalling may also decrease ABCB1 activity [49, 50]. Our data do not suggest that PKCα and/or PKCβ inhibition may play a dominant role in the observed effects on ABCB1 function in our system although some contribution of effects on PKC signalling cannot be excluded, in particular at higher enzastaurin concentrations. We did not observe PKCβ expression in UKF-NB-3^r^VCR^10^ cells. PKCα depletion reduced MARCKS phosphorylation indicating inhibition of PKC signalling but did not affect ABCB1 function. Moreover, the enzastaurin IC_50_ for PKCα inhibition in isolated enzyme assay is 0.8 μM (1) and appears, thus, to be too high to explain effects on ABCB1 function in concentrations as low as 0.3125 μM. Also, enzastaurin-mediated inhibition of PKC signalling became only detectable at a concentration of 1.25 μM after 6 h of incubation or 5 μM after 120 h of incubation. These concentrations are substantially higher than the low enzastaurin concentrations that affected ABCB1-mediated drug transport. In addition, wash-out experiments and determination of ABCB1 ATPase activity demonstrated that the enzastaurin-mediated effects closely resemble those of the ABCB1 substrate verapamil. Therefore, enzastaurin appears to interfere directly with ABCB1, possibly being an ABCB1 substrate. This finding is in concordance with data showing that staurosporine, the lead structure that provided the basis for the synthesis of enzastaurin [1], and staurosporine analogues may interfere with ABCB1 function independently of effects on PKC signalling [43].

In a head-to-head comparison of enzastaurin with staurosporine and its derivatives UCN-01, GF109203X, and RO-31–8220 that had previously been investigated for their interaction with ABCB1-mediated drug transport [43], enzastaurin exerted similar effects on rhodamine 123 accumulation in ABCB1-transduced UKF-NB-3^ABCB1^ cells like staurosporine and UCN-01 that had previously been shown to interfere strongly with ABCB1-mediated drug transport [43]. Also in concert with previous findings [43], GF109203X and RO-31–8220 displayed substantially weaker or no effects on rhodamin 123 accumulation (Suppl. Figure 4A and 4B). Moreover, enzastaurin exerted stronger effects on rhodamin 123 accumulation than verapamil in a direct comparison (Suppl. Figure 4A and 4B).

The notion that enzastaurin interacts directly with ABCB1 is further supported by computational docking studies. Recent analyses had shown that docking studies performed by various approaches are reliable strategies to identify compounds that interact directly with ABCB1 [51–53]. From our previous studies, the docking scores obtained for a group of 54 substrates (including well-known substrates such as ivermectin and cyclosporine) had an average docking score of ≤ −12, like those we observed for enzastaurin in the 3G60 structure, while a group of 69 non-substrates had an average score of ~ −10 kcal/mol using MOE software and the same docking methodology [54, 55].

Notably, the effects of PKC signalling on ABCB1 phosphorylation and function appear to be cell type-dependent. In ovarian carcinoma cells, antisense oligomers directed against PKCα and PKCβ reversed ABCB1-mediated drug resistance [56]. In contrast, PKCβ was not detectable in our model system, and siRNAs targeting PKCα interfered with PKC signalling but not with ABCB1 function. Moreover, p53 was shown to suppress PKCα-mediated ABCB1 activation in leiomyosarcoma, fibrosarcoma, and osteosarcoma cells [35]. In contrast, the enzastaurin-mediated effects on ABCB1 function did not differ between p53 wild-type and p53-mutant neuroblastoma or rhabdomyosarcoma cells in the present study.

In addition to its interaction with ABCB1, enzastaurin interfered with ABCG2-mediated drug transport but the mode of action appears to be different. While enzastaurin stimulated ABCB1 ATPase activity, it inhibited ABCG2 ATPase activity. ABCC1 (also known as MRP1) is another ABC transporter that is known to be of clinical relevance in neuroblastoma [57]. Noteworthy, enzastaurin also sensitised ABCC1-expressing cells to the ABCC1 substrate vincristine and enhanced accumulation of the fluorescent ABCC1 substrate 5-CFDA in ABCC1-expressing cells (Suppl. Figure 5A–5C). Our findings showing that enzastaurin interferes with ABCB1-, ABCG2-, and ABCC1-mediated drug transport are of relevance for the further development of enzastaurin combination therapies. Enzastaurin has been reported to display enhanced activity in combination with various anti-cancer drugs [see e.g. 11, 13, 17, 19, 41; 58–60] including ABCB1, ABCG2, and/or ABCC1 substrates such as paclitaxel [60], docetaxel [58], erlotinib [59], and doxorubicin [11]. In the light of the finding that enzastaurin interferes with the ABCB1-, ABCG2-, and ABCC1-mediated drug transport studies that investigate the combined use of enzastaurin with substrates of these transporters may require careful (re-)evaluation.

In conclusion, our data show that enzastaurin inhibits ABCB1 predominantly through direct interaction independently of effects on PKC signalling or the cellular p53 status. This finding is in particular relevant for the further development of therapies in which enzastaurin is combined with ABCB1 substrates.

## MATERIALS AND METHODS

### Drugs

Enzastaurin was purchased from Selleck Chemicals via BIOZOL GmbH (Eching, Germany). Vincristine was obtained from Sigma-Aldrich Chemie GmbH (Munich, Germany). Rhodamine 123 was purchased from Merck Biosciences (Darmstadt, Germany).

### Cell culture

The MYCN-amplified neuroblastoma cell lines UKF-NB-2 and UKF-NB-3 were established from INSS stage 4 neuroblastoma patients [61, 62]. The rhabdomyosarcoma cell line Rh30 was kindly provided by Dr. P.J. Houghton (St. Jude's Children's Research Hospital, Memphis, TN). The alveolar rhabdomyosarcoma cell line KFR was established from a bone marrow metastasis [63]. K562 cells were obtained from ATCC (Manassas, VA, USA).

The cell lines were adapted to growth in the presence of vincristine 10 ng/ml and named UKF-NB-2^r^VCR^10^ [61], UKF-NB-3^r^VCR^10^ [62], Rh30^r^VCR^10^ [64], KFR^r^VCR^10^ and derived from the RCCL collection (http://www.kent.ac.uk/stms/cmp/RCCL/RCCLabout.html).

The UKF-NB-3 sub-lines expressing ABCB1 (UKF-NB-3^ABCB1^), ABCG2 (also known as BCRP, UKF-NB-3^ABCG2^), or shRNA targeting p53 mRNA (UKF-NB-3^p53shRNA^) were established by lentiviral transfection as described previously [65–68] (http://www.lentigo-vectors.de). ABCB1 expression data for the project cell lines is presented in Suppl. Figure 6.

Fresh neuroblastoma cells (MYCN amplified) were isolated from the bone marrow aspirate of patients with metastasised INSS stage 4 neuroblastoma.

Cells were propagated in IMDM supplemented with 10% foetal calf serum, 100 IU/ml penicillin and 100 mg/ml streptomycin at 37°C.

### Viability assay

Cell viability was tested by the 3-(4,5-dimethylthiazol-2-yl)-2,5-diphenyltetrazolium bromide (MTT) dye reduction assay after 120 h incubation modified as described before [64].

### ABC transporter expression and function

Antibodies directed against ABCB1 (Alexis Biochemicals via AXXORA Deutschland, Lörrach, Germany) or ABCG2 (Kamiya Biomedical Company, Seattle, WA, USA), followed by a secondary antibody labelled with Phycoerythrin (R&D, Wiesbaden, Germany) were used to detect protein expression by flow cytometry (FACSCalibur, BD Biosciences, Heidelberg, Germany).

To investigate ABCB1-mediated drug efflux, cells were pre-incubated with different concentrations of enzastaurin for 30 min. 0.5 μM rhodamine 123 (fluorescent ABCB1 substrate) was added for another 30 min. Then, cell culture medium was removed, cells were washed three times with PBS, and fresh medium containing enzastaurin was added. After another 45 min, cellular fluorescence was analysed by flow cytometry (FACSCalibur). Rhodamine 123 was detected at the FL1 channel.

For wash out kinetic experiments, cells were incubated for 1 h with 0.5 μM rhodamine 123 and enzastaurin at the indicated concentrations. Cells were resuspended in supplemented medium and cellular fluorescence was measured after different time points (t_0_, t_5_, t_15_, t_30_, t_60_, t_120_ minutes) by flow cytometry (FACSCalibur).

To investigate ABCG2-mediated drug efflux, the same procedures were carried out. Mitoxantrone served as fluorescent and cytotoxic ABCG2 substrate. The ABCG2 inhibitor Ko143 [69] was used as control substance.

The ATPase activities of ABCB1 and ABCG2 were determined using membrane preparations (ABCB1-Membran: BD Biosciences, Heidelberg, Germany; ABCG2 membrane: Solvo Biotechnolgy, Budapest, Hungary) and an established kit (BD Biosciences, Heidelberg, Germany) following the manufacturer's instruction.

### PKCα expression

Antibodies directed against PKCα (abcam, Cambridge, UK) followed by a secondary antibody labelled with Phycoerythrin (R&D, Wiesbaden, Germany) were used to detect protein expression by flow cytometry (FACSCalibur, BD Biosciences).

### Western blot

Cells were lysed in Triton X-sample buffer and separated by SDS-PAGE. Proteins were detected using specific antibodies directed against β-actin (BioVision via BioCat GmbH, Heidelberg, Germany), PKCα (abcam, Cambridge, UK), PKCβ (BD Biosciences, Heidelberg, Germany), MARCKS (Cell Signaling Technology, Danvers, MA), and phosphorylated MARCKS (Cell Signaling Technology). Protein bands were visualised by enhanced chemiluminescence using a commercially available kit (Amersham, Freiburg, Germany).

### siRNA transfection experiments

Synthetic siRNA oligonucletides targeting ABCB1 or PRKCA (encoding for PKCα) (ON-TARGET plusSMART pool siRNAs) were purchased from Dharmacon (Lafayette, CO, USA). The non targeting siRNA ON-TARGET plus SMART pool (Dharmacon) was used as negative control. Transfections were performed using the Neon^TM^ Transfection System (Invitrogen, Darmstadt, Germany) according to the manufacturer's protocol. UKF-NB-3^r^VCR^10^ or UKF-NB-3^ABCB1^ cells were grown to about 60–80% confluence, trypsinised and 2 × 10^6^ cells were re-suspended in 200 μl re-suspension buffer containing 2.5 μM siRNA. Electroporation was performed in a pipette tip chamber with previously optimised adjustments (voltage 1400, width 20ms, 2 pulses). After electroporation, the cells were transferred into fibronectin (100 μg/ml)-coated 6-well-plates containing pre-warmed IMDM plus 10% foetal calf serum.

### ABCB1 docking studies

The protein and ligands were prepared for docking in MOE (version 2012.10, Chemical Computing Group Inc., Montreal, Canada). Mouse Abcb1a structures, 3G60, 3G61 and 3G5U [36] were obtained from the protein data bank (http://www.rcsb.org), the human homology model based on this structure from [70]. The crystal parameters were retained and all atoms of ABCB1 were protonated and titrated using default parameters of the software.

To prepare the ligand for docking, atomic charges were initially calculated using Merck Molecular Force Field 94 (MMFF94) force field and then the energy was minimised and atomic charges were re-calculated using Self-Consistent Field (SCF) optimization (AM1 Hamiltonian). Several docking experiments were performed for each protein using the binding site defined by proximity to a co-crystallised ligand and the residues reported by Aller et al. [36] to be involved in the binding pocket of cyclic-tris-(R)-valineselenazole (QZ59-RRR), upper binding pocket of cyclic-tris-(S)-valineselenazole (QZ59-SSS), lower binding pocket of QZ59-SSS, or the verapamil binding site (residues protected from MTS labelling by verapamil) as described by Aller et al [36].

In the MOE dock panel, the placement method was Triangle Matcher, scoring methodology was set to London dG as the first and the second scoring functions, and the final energy was evaluated using the Generalized Born solvation model (GB/VI) and finally, the top five best scoring poses were retained.

Default parameters of the software were used for the calculation of the ligand interactions. These were energy cut-off for H-bonding and ionic interactions at −0.5 kcal/mol and the maximum distance for non-bonded interactions at 4.5 Å. This docking methodology has been validated previously by docking the co-crystallised ligand, QZ59-RRR and comparing the geometries of the ‘docked ABCB1/QZ59-RRR’ structure with the structure of P-gp/QZ59-RRR complexes from x-ray crystallography [71]. The docking methodology using MOE has a built in conformational search that conducts a systematic search covering all combinations of angles on a grid if this will result in under 5000 conformers. Otherwise a stochastic sampling of conformations is conducted. In addition to this automatic conformational search in one of the docking experiments for each protein, we performed a prior conformational analysis before the docking and used all the resulting conformations. MOE conformational search was used with LowModeMD sampling method. This sampling method has been suggested as the method of choice for larger flexible compounds and macrocycles [70]. Using the default settings of the software, 74 different conformations were generated and used in one docking experiment for each of the proteins.

### Statistics

Results are expressed as mean ± S.D. of at least three experiments. Comparisons between two groups were performed using Student's *t*-test. Three and more groups were compared by ANOVA followed by the Student-Newman-Keuls test. *P* values lower than 0.05 were considered to be significant.

## SUPPLEMENTARY FIGURES AND TABLES



## Abbreviations

ABC transporter: ATP-binding cassette transporter
MARCKS: myristoylated alanine-rich C-kinase substrate
MTT: 3-(4,5-dimethylthiazol-2-yl)-2,5-diphenyltetrazolium bromide
RCCL collection: Resistant Cancer Cell Line collection
