# Supplementary material for: Enzastaurin inhibits ABCB1-mediated drug efflux independently of effects on protein kinase C signalling and the cellular p53 status
Source: Oncotarget. 2015 Feb 13;6(19):17605–20. doi: 10.18632/oncotarget.2889 (PMC4627332; doi:10.18632/oncotarget.2889)
Supplement: Supplementary file 1 [file oncotarget-06-17605-s001.pdf]

SUPPLEMENTARY FIGURES AND TABLES

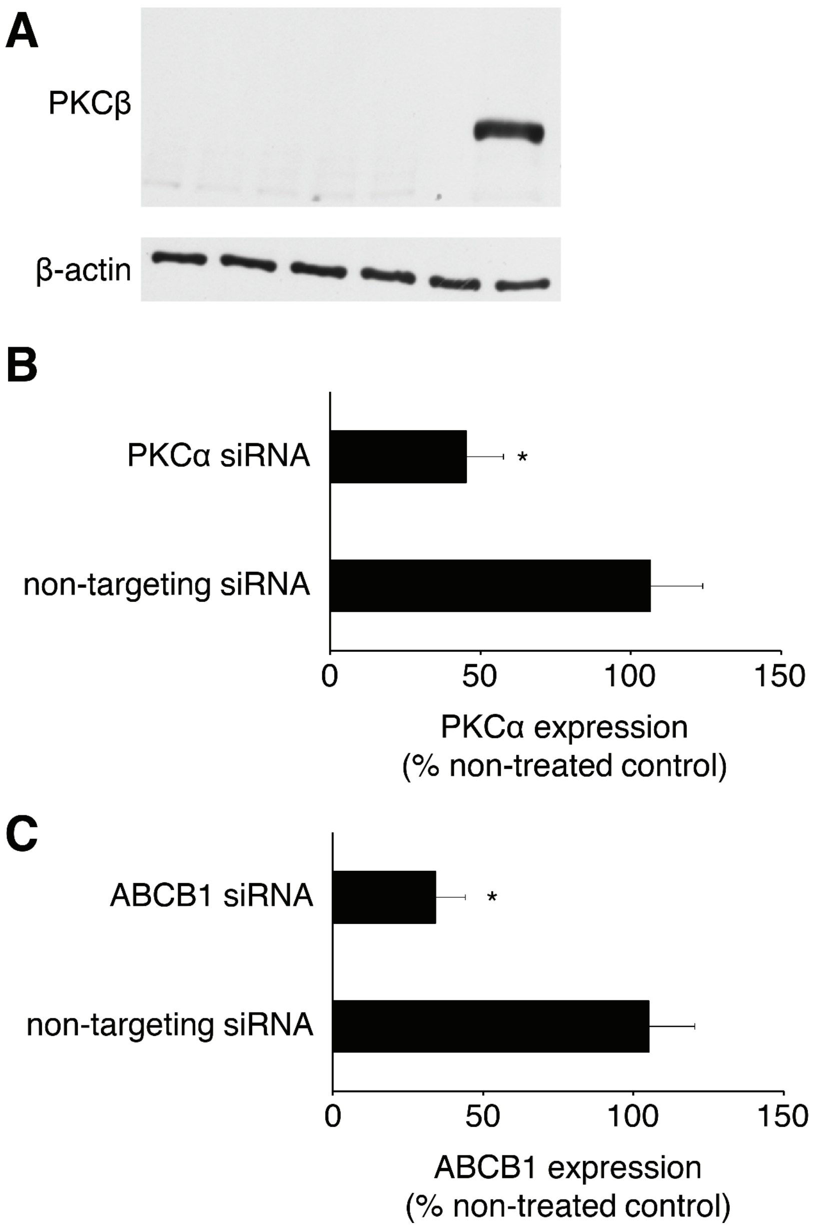

Supplementary Figure 1: Absence of detectable PKCβ protein levels and PKCα and ABCB1 depletion in UKF-NB-3^{r}VCR^{10} cells. **(A)** A Western blot for PKCβ did not result in detectable PKCβ protein levels in UKF-NB-3^{r}VCR^{10} cells although PKCβ was readily detectable in K562 cells. **(B)** siRNA-mediated depletion of PKCα or ABCB1 in UKF-NB-3^{r}VCR^{10} cells 48 h post- transfection determined by flow cytometry using specific antibodies. Values are means ± S.D. (*$P < 0.05$) relative to non-targeting siRNA as indicated by *t*-test.

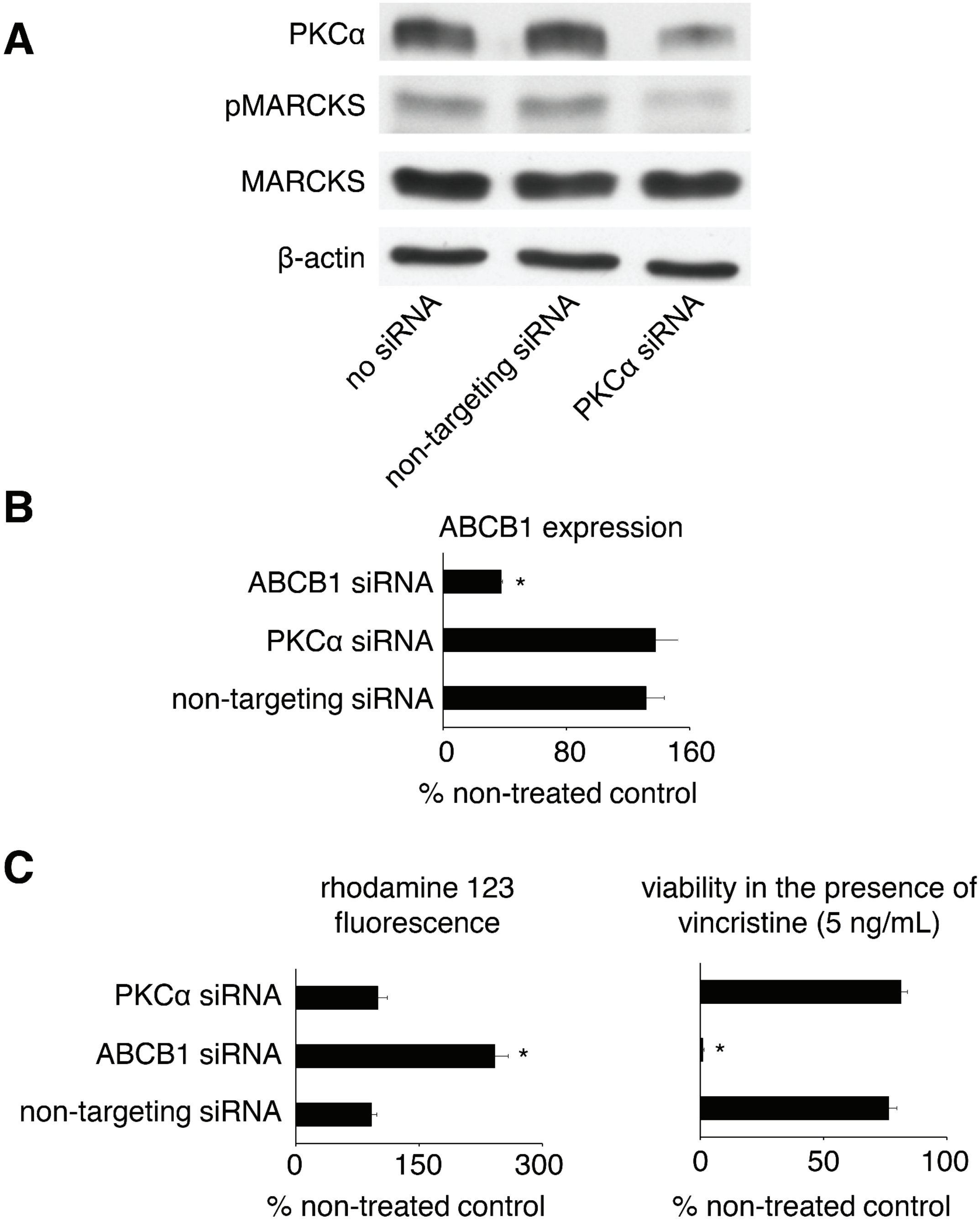

Supplementary Figure 2: Effects of siRNA-mediated PKCα depletion on PKC signalling as indicated by MARCKS phosphorylation and on ABCB1 function. **(A)** Effects of siRNA- mediated PKCα depletion on MARCKS phosphorylation determined in UKF-NB-3^{ABCB1} cells 48 h after transfection; **(B)** siRNA-mediated PKCα depletion does not affect ABCB1 expression. **P* < 0.05 relative to non-treated control; **(C)** siRNA directed against ABCB1 (but not siRNA directed against PKCα) increases (1) accumulation of the fluorescent ABCB1 substrate rhodamine 123 (0.5 µM) in ABCB1-expressing UKF-NB-3^{ABCB1} cells and (2) the sensitivity of UKF-NB-3^{ABCB1} cells to the cytotoxic ABCB1 substrate vincristine. **P* < 0.05 relative to non-targeting siRNA.

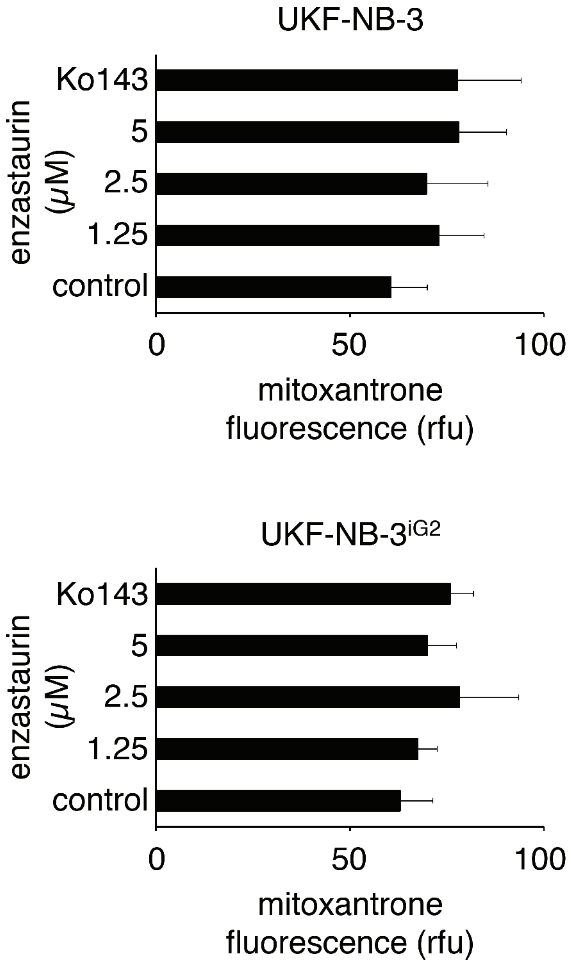

Supplementary Figure 3: Influence of enzastaurin on accumulation of mitoxantrone (40 µM; a fluorescent ABCG2 substrate) in non-ABCG2-expressing UKF-NB-3 cells and UKF-NB-3 cells transduced with a control vector (UKF-NB-3^{iG2}) as detected by flow cytometry (RFU = relative fluorescence units). The ABCG2 inhibitor Ko143 (1 µM) served as positive control.

Cell line	Drug	IC_{50} (nM)
UKF-NB-3[ABCB1]	staurosporine	9.80 ± 2.49
	UCN-01	104.6 ± 23.7
	enzastaurin	8,365 ± 1,812
	GF109203X	5,244 ± 1,408
	RO-31-8220	4,996 ± 1950
	verapamil	45,606 ± 10,129
UKF-NB-3[Cer2]	staurosporine	3.35 ± 0.98
	UCN-01	77.2 ± 20.3
	enzastaurin	7,862 ± 1,041
	GF109203X	2,477 ± 604
	RO-31-8220	972 ± 195
	verapamil	44,157 ± 9,211

Supplementry Figure 4A: Effects of staurosporine, enzastaurin, UCN-01, GF109203X, and RO-31-8220, and verapamil on neuroblastoma cell viability.

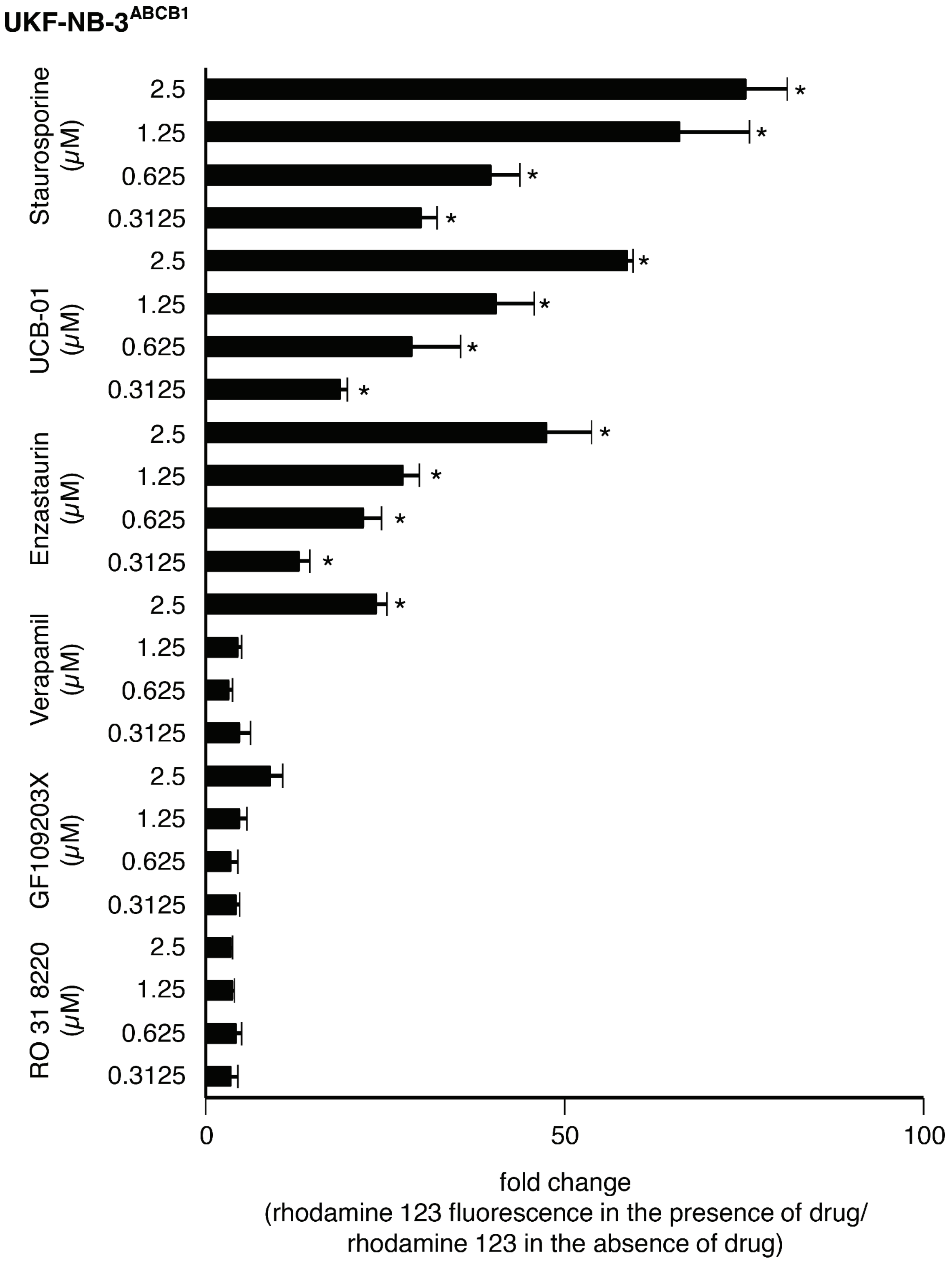

Supplementary Figure 4B: (*Continued*) Effects of staurosporine, enzastaurin, UCN-01, GF109203X, RO-31-8220, and verapamil on accumulation of the fluorescent ABCB1 substrate rhodamin 123 in ABCB1-transduced UKF-NB-3 cells (UKF-NB-3ABCB1) and UKF-NB-3 cells transduced with a control vector (UKF-NB-3Cer2). The investigated drug concentrations did not affect cell viability in this short-term assay as indicated by MTT assay (data not shown). Results are expressed as fold change (rhodamine 123 fluorescence in the presence of drug/ rhodamine 123 in the absence of drug). $^*P < 0.05$ relative to rhodamine 123 alone.

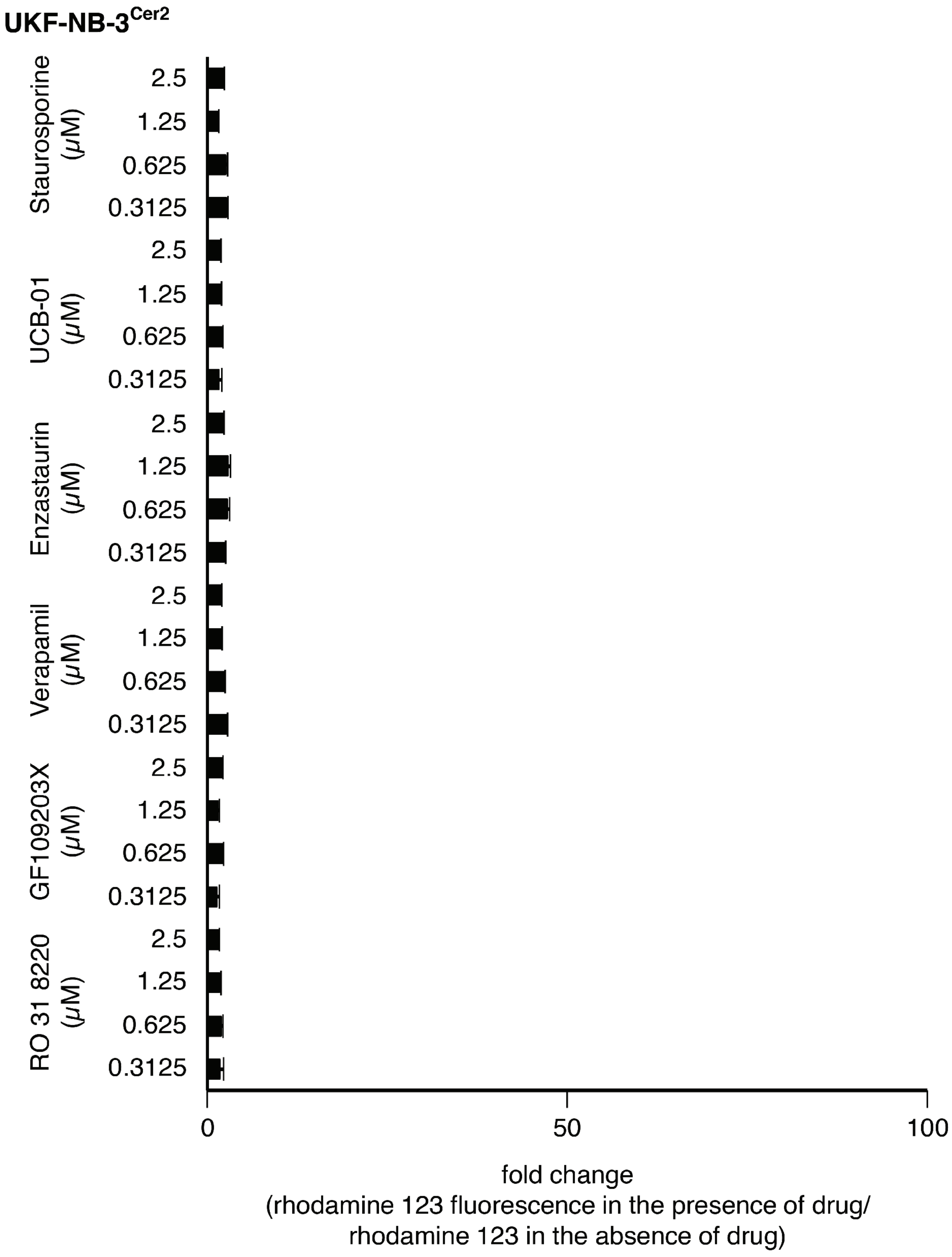

Supplementary Figure 4C: (*Continued*) Effects of staurosporine, enzastaurin, UCN-01, GF109203X, RO-31-8220, and verapamil on accumulation of the fluorescent ABCB1 substrate rhodamin 123 in ABCB1-transduced UKF-NB-3 cells (UKF-NB-3^{ABCB1}) and UKF-NB-3 cells transduced with a control vector (UKF-NB-3^{Cer2}). The investigated drug concentrations did not affect cell viability in this short-term assay as indicated by MTT assay (data not shown). Results are expressed as fold change (rhodamine 123 fluorescence in the presence of drug/ rhodamine 123 in the absence of drug). *$P < 0.05$ relative to rhodamine 123 alone.

1) NLF^{r}VCR^{10} displays enhanced expression of ABCC1 relative to NLF but not of ABCB1 or ABCG2 (* P < 0.05 relative to NLF).

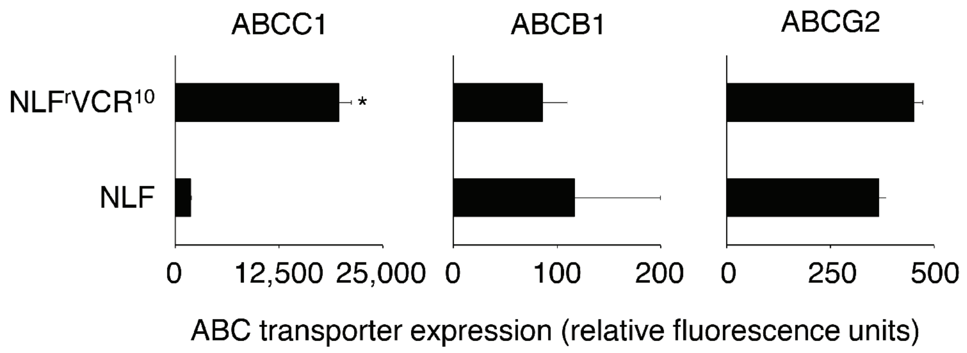

2) The ABCC1 inhibitors MK571 (10μM) and verapamil (5μM) sensitise NLF^{r}VCR^{10} cells but not NLF cells to the ABCC1 substrate vincristine. MK571 or verapamil alone did not affect the viability of the investigated cells (* P < 0.05 relative to vincristine alone (control)).

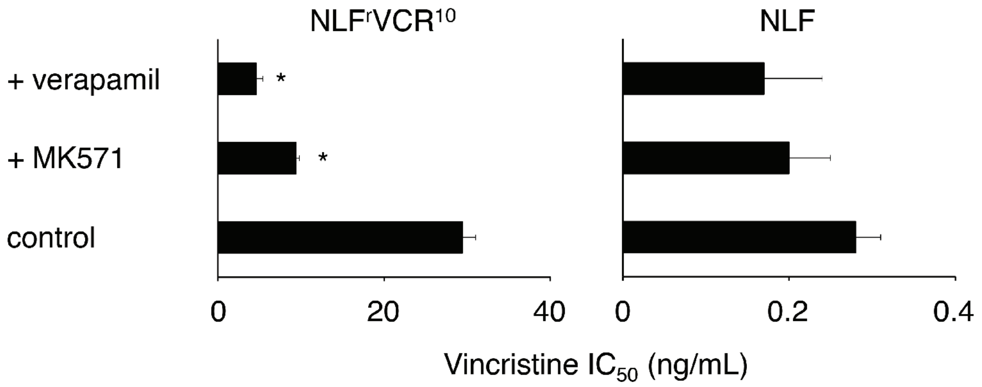

3) SiRNA directed against ABCC1 sensitises NLF^{r}VCR^{10} cells to vincristine (scr = scrambled non-targeting siRNA; * P < 0.05 relative to no siRNA).

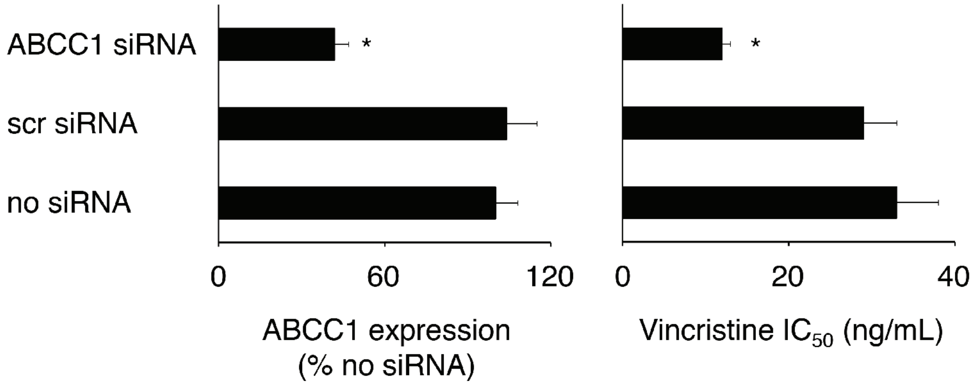

Supplementary Figure 5A: NLF^{r}VCR^{10} as ABCC1 model. (1) NLF^{r}VCR^{10} displays enhanced expression of ABCC1 relative to NLF but not of ABCB1 or ABCG2 (**P* < 0.05 relative to NLF). **(2)** The ABCC1 inhibitors MK571 (10 µM) and verapamil (5 µM) sensitise NLF^{r}VCR^{10} cells but not NLF cells to the ABCC1 substrate vincristine. MK571 or verapamil alone did not affect the viability of the investigated cells (**P* < 0.05 relative to vincristine alone (control)). **(3)** SiRNA directed against ABCC1 sensitises NLF^{r}VCR^{10} cells to vincristine (scr = scrambled non-targeting siRNA; **P* < 0.05 relative to no siRNA). (*Continued*)

NLF[r]VCR[10]

Enzastaurin (μM)	Viability in the presence of enzastaurin alone	vincristine IC_{50} (ng/mL)	fold change*
0	100	36.44 ± 2.25	
0.3125	106 ± 8	24.10 ± 1.08	1.51
0.625	98 ± 6	22.71 ± 2.21	1.60
1.25	96 ± 6	15.52 ± 3.84	2.35
2.5	88 ± 5	8.92 ± 0.77	4.09
5	52 ± 1	2.03 ± 0.33	17.95

G62

Enzastaurin (μM)	Viability in the presence of enzastaurin alone	vincristine IC_{50} (ng/mL)	fold change
0	100	1.95 ± 0.25	
0.3125	94 ± 4	1.61 ± 0.10	1.21
0.625	89 ± 4	1.51 ± 0.11	1.29
1.25	80 ± 2	1.09 ± 0.03	1.79
2.5	73 ± 3	0.61 ± 0.07	3.20
5	55 ± 4	0.24 ± 0.06	8.13

PC3[r]VCR[20]

Enzastaurin (μM)	Viability in the presence of enzastaurin alone	vincristine IC_{50} (ng/mL)	fold change
0	100	20.23 ± 1.41	
0.3125	84 ± 7	15.72 ± 4.44	1.29
0.625	80 ± 5	13.80 ± 4.23	1.47
1.25	71 ± 1	9.12 ± 1.95	2.22
2.5	62 ± 3	5.14 ± 0.26	3.94
5	32 ± 1	5.16 ± 1.30	3.92

* IC_{50} vincristine/ IC_{50} vincristine in the presence of enzastaurin

Supplementary Figure 5B: (*Continued*) Enzastaurin sensitises ABCC1-expressing NLF[r]VCR[10], G62, and PC3[r]VCR[10] cells to the ABCC1 substrate vincristine. Cell viability was determined by MTT assay after 120 h of incubation (IC_{50} = concentration that reduces cell viability by 50%). (*Continued*)

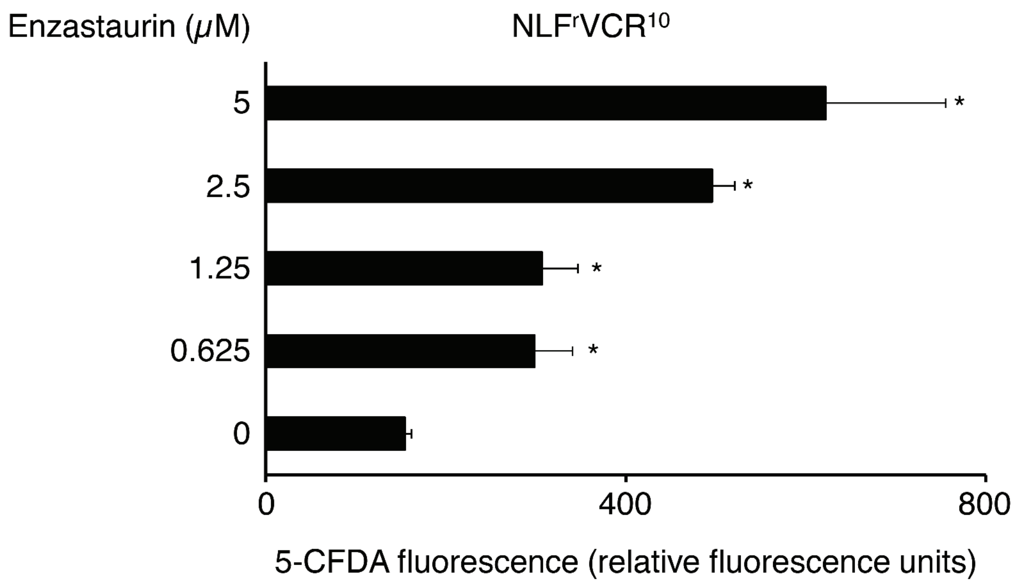

Representative flow cytometry histograms

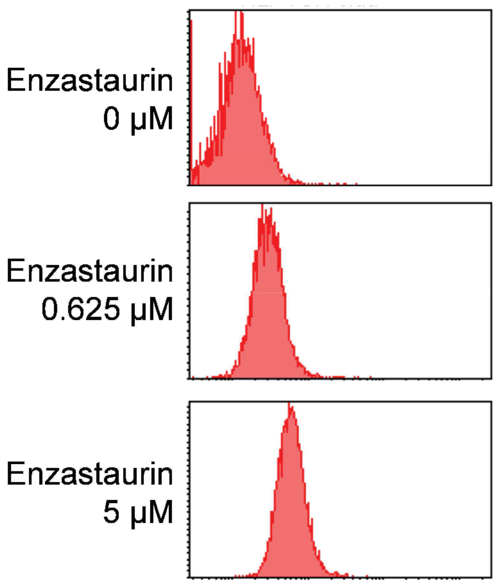

Supplementary Figure 5C: (*Continued*) Effects of enzastaurin on the accumulation of the fluorescent ABCC1 substrate 5-CFDA in ABCC1-expressing NLF^{r}VCR^{10} cells (*$P < 0.05$ relative to non-treated control).

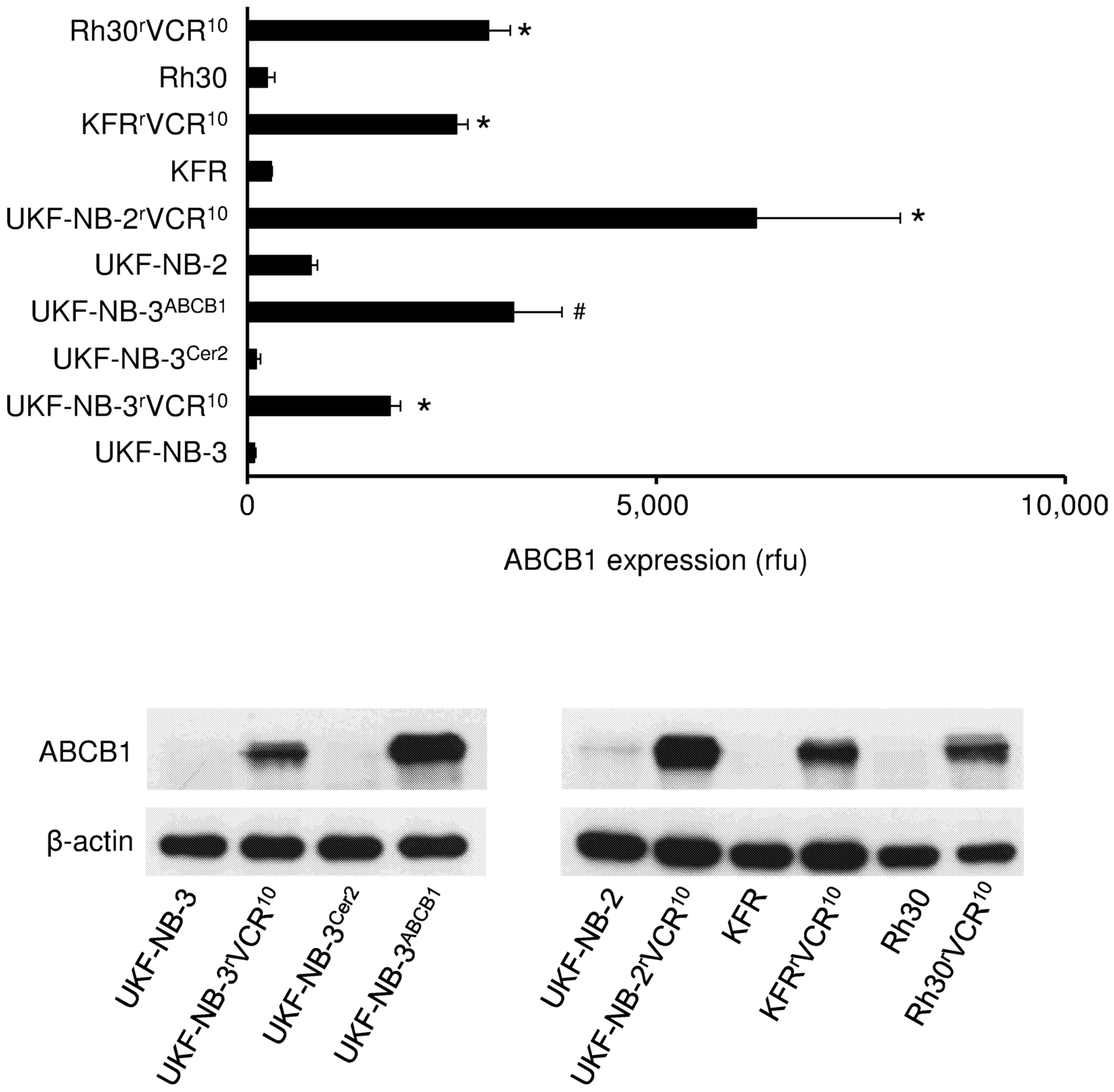

Supplementary Figure 6: ABCB1 expression in the project cell lines as determined by flow cytometry or Western blot; *P < 0.05 relative to respective parental cell line, # P < 0.05 UKF-NB-3^{Cer2}.

Supplementary Table 1: Influence of enzastaurin (1.25 µM) on the vincristine sensitivity of neuroblastoma and rhabdomyosarcoma cells. Cell viability was determined after a 5 day incubation period by MTT assay. Concentrations that reduced cell viability by 50% (IC_{50}) were calculated.

Cell line	ABCB1status	IC_{50}[1] vincristine (ng/ml)	Influence of enzastaurin on the vincristine IC_{50}: cell viability enzastaurin (% control)	IC_{50} vincristine (ng/ml)	Fold change[2]
UKF-NB-3	−	0.29 ± 0.09	99.5 ± 15.0	0.30 ± 0.16	0.97
UKF-NB-3^{r}VCR^{10}	+	53.0 ± 7.7	85.3 ± 12.2	1.69 ± 0.41[3]	31.37
UKF-NB-3^{ABCB1}	+	33.0 ± 8.2	99.9 ± 12.3	0.77 ± 0.11[3]	42.86
UKF-NB-3^{Cer2}	−	0.56 ± 0.06	91.7 ± 10.9	0.52 ± 0.08	1.08
UKF-NB-2	−	0.61 ± 0.28	95.6 ± 19.8	0.49 ± 0.22	1.24
UKF-NB-2^{r}VCR^{10}	+	50.6 ± 9.0	88.3 ± 9.6	2.08 ± 0.39[3]	24.34
KFR	−	0.47 ± 0.11	97.4 ± 13.7	0.26 ± 0.04	1.81
KFR^{r}VCR^{10}	+	51.6 ± 13.2	106.8 ± 11.2	2.46 ± 0.37[3]	20.99
Rh30	−	0.38 ± 0.01	108.1 ± 11.0	0.40 ± 0.18	0.95
Rh30^{r}VCR^{10}	+	76.7 ± 14.5	104.6 ± 8.2	1.97 ± 0.28[3]	38.86

[1]Values are mean ± S.D. of three independent experiments.
[2]IC_{50} vincristine/ IC_{50} vincristine in the presence of enzastaurin.
[3]*P* < 0.05 relative to IC_{50} vincristine in the absence of enzastaurin as indicated by *t*-test.

Supplementary Table 2: Influence of enzastaurin on the sensitivity of ABCB1-expressing UKF-NB-3^{r}VCR^{10} cells to the cytotoxic ABCB1 substrates paclitaxel and actinomycin D. Cell viability was determined after a 5 day incubation period by MTT assay. Concentrations that reduced cell viability by 50% (IC_{50}) were calculated.

Enzastaurin	IC_{50}[1] paclitaxel (ng/ml)	Influence of enzastaurin on the paclitaxel IC_{50}: cell viability enzastaurin (% control)	IC_{50} paclitaxel (ng/ml)	Fold sensitisation[2]
0.625 µM	75.31 ± 8.36	87.74 ± 9.18	5.62 ± 0.74[3]	13.4
1.25 µM	91.00 ± 10.27	87.96 ± 8.83	3.04 ± 0.41[3]	29.93

Enzastaurin	IC_{50}[4] actinomycin D (ng/ml)	Influence of enzastaurin on the actinomycin D IC_{50}: cell viability enzastaurin (% control)	IC_{50} actinomycin D (ng/ml)	Fold sensitisation[5]
0.625 µM	2.10 ± 0.27	90.30 ± 6.38	0.38 ± 0.05[6]	5.53
1.25 µM	2.28 ± 0.15	88.23 ± 4.16	0.32 ± 0.04[6]	7.13

[1]Values are mean ± S.D. of three independent experiments.
[2]IC_{50} paclitaxel/ IC_{50} paclitaxel in the presence of enzastaurin.
[3]*P* < 0.05 relative to IC_{50} paclitaxel in the absence of enzastaurin as indicated by *t*-test.
[4]Values are mean ± S.D. of three independent experiments.
[5]IC_{50} actinomycin D/ IC_{50} actinomycin D in the presence of enzastaurin.
[6]*P* < 0.05 relative to IC_{50} actinomycin D in the absence of enzastaurin as indicated by *t*-test.

Supplementary Table 3A: Concentration-dependent influence of enzastaurin on the sensitivity of low ABCB1-expressing UKF-NB-3 and high ABCB1-expressing UKF-NB-3rVCR10 neuroblastoma cells to the ABCB1 substrate vincristine. Cell viability was determined after a 5 day incubation period by MTT assay. Concentrations that reduced cell viability by 50% (IC_{50}) were calculated.

		Influence of enzastaurin on the vincristine IC_{50} in UKF-NB-3 cells (low ABCB1)		
**Enzastaurin (µM)**	**IC_{50}[1] vincristine (ng/ml)**	**cell viability enzastaurin (% control)**	**IC_{50} vincristine (ng/ml)**	**Fold sensitisation[2]**
0.3125	0.25 ± 0.05	103.45 ± 12.22	0.23 ± 0.07	1.09
0.625	0.22 ± 0.08	91.84 ± 10.73	0.31 ± 0.04	0.71
1.25	0.33 ± 0.10	86.91 ± 11.79	0.21 ± 0.08	1.57
2.5 µM	0.27 ± 0.06	74.29 ± 9.18	0.20 ± 0.04	1.35

		Influence of enzastaurin on the vincristine IC_{50} in UKF-NB-3rVCR10 cells (high ABCB1)		
**Enzastaurin (µM)**	**IC_{50}[1] vincristine (ng/ml)**	**cell viability enzastaurin (% control)**	**IC_{50} vincristine (ng/ml)**	**Fold sensitisation[2]**
0.3125	45.21 ± 6.09	95.35 ± 9.93	8.93 ± 1.16[3]	5.06
0.625	47.27 ± 5.89	100.92 ± 14.51	4.43 ± 0.55[3]	10.67
1.25	53.02 ± 7.68	85.32 ± 12.17	1.69 ± 0.41[3]	31.37
2.5 µM	56.51 ± 6.75	65.71 ± 9.74	0.60 ± 0.12[3]	94.18

[1]Values are mean ± S.D. of three independent experiments.
[2]IC_{50} vincristine/ IC_{50} vincristine in the presence of enzastaurin.
[3]$P < 0.05$ relative to IC_{50} vincristine in the absence of enzastaurin as indicated by *t*-test.

Supplementary Table 3B: (*Continued*) Concentration-dependent influence of enzastaurin on the sensitivity of low ABCB1-expressing UKF-NB-2 and high ABCB1-expressing UKF-NB-2^{r}VCR^{10} neuroblastoma cells to the ABCB1 substrate vincristine. Cell viability was determined after a 5 day incubation period by MTT assay. Concentrations that reduced cell viability by 50% (IC_{50}) were calculated.

		Influence of enzastaurin on the vincristine IC_{50} in UKF-NB-2 cells (low ABCB1)		
**Enzastaurin (µM)**	**IC_{50}[1] vincristine (ng/ml)**	**cell viability enzastaurin (% control)**	**IC_{50} vincristine (ng/ml)**	**Fold sensitisation[2]**
0.3125	0.55 ± 0.16	91.52 ± 17.21	0.57 ± 0.12	0.96
0.625	0.65 ± 0.11	94.48 ± 8.13	0.54 ± 0.10	1.20
1.25	0.53 ± 0.09	72.32 ± 9.95	0.48 ± 0.06	1.10
2.5 µM	0.56 ± 0.13	58.13 ± 8.37	0.45 ± 0.04	1.24

		Influence of enzastaurin on the vincristine IC_{50} in UKF-NB-2^{r}VCR^{10} cells (high ABCB1)		
**Enzastaurin (µM)**	**IC_{50}[1] vincristine (ng/ml)**	**cell viability enzastaurin (% control)**	**IC_{50} vincristine (ng/ml)**	**Fold sensitisation[2]**
0.3125	57.98 ± 10.73	93.00 ± 10.26	11.86 ± 1.71[3]	4.89
0.625	40.80 ± 8.43	90.97 ± 6.21	5.72 ± 1.13[3]	7.13
1.25	50.62 ± 8.97	88.28 ± 9.61	2.08 ± 0.39[3]	24.34
2.5 µM	50.09 ± 6.92	69.81 ± 7.86	0.46 ± 0.09[3]	108.41

[1]Values are mean ± S.D. of three independent experiments.
[2]IC_{50} vincristine/ IC_{50} vincristine in the presence of enzastaurin.
[3]$P < 0.05$ relative to IC_{50} vincristine in the absence of enzastaurin as indicated by *t*-test.

Supplementary Table 3C: (*Continued*) Concentration-dependent influence of enzastaurin on the sensitivity of low ABCB1-expressing KFR and high ABCB1-expressing KFR^{r}VCR^{10} rhabdomyosarcoma cells to the ABCB1 substrate vincristine. Cell viability was determined after a 5 day incubation period by MTT assay. Concentrations that reduced cell viability by 50% (IC_{50}) were calculated.

		Influence of enzastaurin on the vincristine IC_{50} in KFR cells (low ABCB1)		
**Enzastaurin (µM)**	**IC_{50} vincristine[1] (ng/ml)**	**cell viability enzastaurin (% control)**	**IC_{50} vincristine (ng/ml)**	**Fold sensitisation[2]**
0.3125	0.54 ± 0.12	101.13 ± 6.27	0.60 ± 0.09	0.90
0.625	0.59 ± 0.15	107.91 ± 11.84	0.55 ± 0.08	1.07
1.25	0.47 ± 0.11	97.37 ± 13.72	0.26 ± 0.04	1.81
2.5 µM	0.54 ± 0.16	88.61 ± 7.99	0.27 ± 0.09	2.00

		Influence of enzastaurin on the vincristine IC_{50} in KFR^{r}VCR^{10} cells (high ABCB1)		
**Enzastaurin (µM)**	**IC_{50}[1] vincristine (ng/ml)**	**cell viability enzastaurin (% control)**	**IC_{50} vincristine (ng/ml)**	**Fold sensitisation[2]**
0.3125	47.10 ± 6.24	108.29 ± 13.41	20.50 ± 2.15[3]	2.30
0.625	48.74 ± 2.81	111.34 ± 7.26	5.90 ± 1.96[3]	8.25
1.25	51.63 ± 13.18	108.83 ± 11.27	2.46 ± 0.37[3]	20.99
2.5 µM	52.44 ± 8.50	84.28 ± 8.82	0.93 ± 0.25[3]	56.27

[1]Values are mean ± S.D. of three independent experiments.
[2]IC_{50} vincristine/ IC_{50} vincristine in the presence of enzastaurin.
[3]$P < 0.05$ relative to IC_{50} vincristine in the absence of enzastaurin as indicated by *t*-test.

Supplementary Table 3D: (*Continued*) Concentration-dependent influence of enzastaurin on the sensitivity of low ABCB1-expressing Rh30 and high ABCB1-expressing $Rh30^{r}VCR^{10}$ rhabdomyosarcoma cells to the ABCB1 substrate vincristine. Cell viability was determined after a 5 day incubation period by MTT assay. Concentrations that reduced cell viability by 50% (IC_{50}) were calculated.

		Influence of enzastaurin on the vincristine IC_{50} in Rh30 cells (low ABCB1)		
**Enzastaurin (µM)**	**IC_{50}[1] vincristine (ng/ml)**	**cell viability enzastaurin (% control)**	**IC_{50} vincristine (ng/ml)**	**Fold sensitisation[2]**
0.3125	0.33 ± 0.05	88.79 ± 13.73	0.30 ± 0.04	1.10
0.625	0.23 ± 0.03	97.40 ± 8.91	0.31 ± 0.03	0.74
1.25	0.38 ± 0.01	108.08 ± 11.00	0.40 ± 0.18	0.95
2.5 µM	0.37 ± 0.04	97.41 ± 1.24	0.25 ± 0.05	1.46

		Influence of enzastaurin on the vincristine IC_{50} in $Rh30^{r}VCR^{10}$ cells (high ABCB1)		
**Enzastaurin (µM)**	**IC_{50}[1] vincristine (ng/ml)**	**cell viability enzastaurin (% control)**	**IC_{50} vincristine (ng/ml)**	**Fold sensitisation[2]**
0.3125	71.13 ± 8.46	92.98 ± 10.76	11.40 ± 1.21[3]	6.24
0.625	77.88 ± 4.10	113.21 ± 11.09	8.13 ± 1.27[3]	9.58
1.25	76.68 ± 14.54	104.63 ± 8.21	1.97 ± 0.28[3]	38.86
2.5 µM	68.45 ± 15.37	91.82 ± 8.20	0.48 ± 0.30[3]	142.61

[1]Values are mean ± S.D. of three independent experiments.
[2]IC_{50} vincristine/ IC_{50} vincristine in the presence of enzastaurin.
[3]$P < 0.05$ relative to IC_{50} vincristine in the absence of enzastaurin as indicated by *t*-test.

Supplementary Table 4: Protein interaction energies (kcal/mol) of the top five scoring poses for the docking of enzastaurin into the different binding pockets of several ABCB1 structures; the best interaction energies are highlighted in bold

ABCB1 Structure	Docking energy of the five top scoring poses (left to right)					Average	Binding site used
3G60	**−14.04**	−12.74	−12.71	−12.25	−11.76	**−12.70**	Upper QZ59-SSS binding site
3G60	**−13.15**	−12.96	−12.86	−12.80	−12.71	**−12.90**	Lower QZ59-SSS binding residues
3G60	**−16.60**	−12.81	−12.38	−11.61	−11.47	**−12.97**	QZ59-RRR binding residues
3G60	−12.33	−12.77	−12.52	−12.27	−11.70	−12.32	QZ59-RRR and upper and lower QZ59-SSS residues
3G60	−12.62	−12.56	−11.95	−11.90	−11.63	−12.13	Verapamil binding residues
3G60	**−15.79**	−13.65	−13.03	−12.93	−12.72	**−13.62**	Lower QZ59-SSS binding site[a]
3G60	−11.20	−11.00	−10.87	−10.23	−10.09	−10.68	QZ59-SSS upper and lower and QZ59-RRR residues[a]
3G61	−10.39	−9.99	−9.84	−9.76	−9.66	−9.93	Lower QZ59-SSS binding site[a]

(*Continued*)

ABCB1 Structure	Docking energy of the five top scoring poses (left to right)					Average	Binding site used
3G61	**−13.43**	−13.24	−12.72	−12.20	−11.90	**−12.70**	Upper QZ59-SSS binding site
3G61	−11.14	−10.81	−10.37	−9.98	−9.77	−10.41	Lower QZ59-SSS binding site
3G61	−12.33	−12.17	−11.78	−11.36	−10.81	−11.69	QZ59-SSS upper and lower and QZ59-RRR residues
3G61	−10.71	−10.01	−9.89	−9.82	−9.30	−9.95	Verapamil binding residues
3G61	−9.66	−8.53	−8.53	−8.33	−8.01	−8.61	QZ59-RRR binding residues
3G61	−12.80	−12.74	−12.58	−12.55	−12.50	**−12.63**	QZ59-SSS upper and lower and QZ59-RRR residues[a]
3G5U	−10.58	−9.79	−9.36	−9.00	−8.99	−9.54	Upper and lower QZ59-SSS residues
3G5U	**−12.98**	−12.85	−11.95	−10.52	−10.35	−11.73	QZ59-SSS upper and lower and QZ59-RRR residues
3G5U	−11.89	−11.64	−11.23	−11.08	−10.87	−11.34	QZ59-SSS upper and lower and QZ59-RRR residues[a]
3G5U	−9.01	−8.95	−8.93	−8.76	−8.59	−8.85	Verapamil binding residues
3G5U	**−13.67**	−12.20	−10.51	−10.43	−10.12	−11.39	QZ59-RRR binding residues
Human	−12.06	−11.96	−11.39	−10.59	−10.13	−11.23	Upper and Lower QZ59-SSS binding residues
Human	**−13.30**	−12.37	−9.28	−9.18	−9.01	−10.63	Upper and Lower QZ59-SSS binding and QZ59-RRR residues
Human	−11.84	−10.75	−10.22	−9.80	−9.77	−10.48	QZ59-SSS upper and lower and QZ59-RRR residues[a]
Human	−10.42	−9.58	−9.07	−9.05	−8.93	−9.41	Verapamil binding residues
Human	−11.37	−10.35	−10.22	−9.77	−9.35	−10.21	QZ59-RRR binding residues

[a]Conformational search was performed prior to docking and 100 docking poses were retained.

Supplementary Table S5: Ligand interaction report for the interaction of enzastaurin with ABCB1 binding sites listing the important interactions for the top poses

ABCB1 Structure	Pose	Ligand	Atom/group in residue/	interaction	Distance (Å)	E (kcal/mol)	Binding site used
3G60	1	C8 21	6-ring/ Phe728	H-pi	4.26	−1.1	Upper and Lower QZ59-SSS binding site
	2	6–6ing	CG2/ Val978	pi-H	3.82	−0.6	
3G60	1	6-ring	6-ring/ Phe724	pi-pi	3.58	−0.0	Lower QZ59-SSS binding residues
	2	6-ring	6-ring/ Phe974	pi-pi	3.71	−0.0	
	3	C 29	SD/ Met68	H-donor	4.49	−0.7	
	3	5-ring	6-ring/ Phe728	pi-pi	3.83	−0.0	
3G60	1	5-ring	6-ring/ Phe71	pi-pi	3.89	−0.0	QZ59-RRR binding residues
	1	6-ring	6-ring/ Phe71	pi-pi	3.23	−0.0	
	2	5-ring	6-ring/ Phe728	pi-pi	3.78	−0.0	
3G60	1	N 15	OE1/ Gln986	H-donor	2.69	−1.5	QZ59-RRR and upper and lower QZ59-SSS residues
3G60	1	6-ring	6-ring/ Phe974	pi-pi	3.91	−0.0	Verapamil binding residues
3G60	1	N15	OG/ Ser975	H-donor	2.7	−1.1	Upper and Lower QZ59-SSS binding site[a]
	1	6-ring	CA/ Gln721	pi-H	4.06	−0.7	
	2	5-ring	CG2/ Val978	pi-H	3.96	−0.6	
	2	6-ring	6-ring/ Phe728	pi-pi	3.48	−0.0	
3G60	1	C 32	6-ring/ Tyr303	H-pi	3.72	−0.6	QZ59-SSS upper and lower and QZ59-RRR residues[a]
	2	N 15	OE1/ Gln721	H-donor	3.49	−0.7	
	2	C 29	6-ring/ Phe299	H-pi	4.58	−0.6	
	2	C 31	6-ring/ Tyr303	H-pi	4.06	−0.6	
	2	5-ring	CD1/Leu300	pi-H	4.02	−0.6	
3G61	1	O 17	ND2/ Asn717	H-acceptor	2.78	−1.3	Upper and Lower QZ59-SSS binding site[a]
	2	N 15	6-ring/ Phe728	H-pi	4.27	−1.5	
	2	5-ring	CZ/ Phe332	pi-H	3.86	−0.6	
	2	5-ring	6-ring/ Phe974	pi-pi	3.88	−0.0	
3G61	1	N 30	6-ring/ Phe974	cation-pi	3.4	−2	Upper QZ59-SSS binding site
	1	C 31	6-ring/ Phe728	H-pi	4.5	−0.7	
	1	5-ring	6-ring/ Phe953	pi-pi	3.75	0	
3G61	1	O 17	ND2/ Asn717	H-acceptor	2.92	−1	Lower QZ59-SSS binding site
3G61	1	5-ring	CD2/ Tyr494	pi-H	4.39	−1.2	QZ59-SSS upper and lower and QZ59-RRR residues
	1	6-ring	6 ring/ Phe974	pi-pi	3.75	−0.0	

(*Continued*)

ABCB1 Structure	Pose	Ligand	Atom/group in residue/	interaction	Distance (Å)	E (kcal/mol)	Binding site used
	2	N 15	SD/ Met67	H-donor	3.9	−3	
	2	N 30	6-ring/ Phe974	caton-pi	3.45	−1.8	
	2	5-ring	6-ring/ Phe953	pi-pi	3.73	−0.0	
	2	6-ring	6-ring/ Phe953	pi-pi	3.9	−0.0	
3G61	1	5-ring	6-ring/ Phe953	pi-pi	3.72	−0.0	Verapamil binding residues
	2	C 31	6-ring/ Phe974	H-pi	4.58	−1	
	2	6-ring	6-ring/ Phe728	pi-pi	3.66	−0.0	
3G61	1	6-ring	CA/ Gly342	pi-H	4.34	−0.6	QZ59-RRR binding residues
	2	N 23	6-ring/ Phe728	H-pi	4.06	−2.2	
	2	5-ring	6-ring/ Phe974	pi-pi	3.7	−0.0	
3G61	1	6-ring	CE2/ Phe332	pi-H	3.56	−0.7	QZ59-SSS upper and lower and QZ59-RRR residues[a]
	2	6-ring	CA/ Phe974	pi-H	3.61	−0.6	
	2	5-ring	6-ring/ Phe71	pi-pi	3.64	−0.0	
	2	6-ring	6-ring/ Phe71	pi-pi	3.55	−0.0	
3G5U	1	6-ring	NE2/ Gln191	pi-H	3.6	−1.9	Upper and lower QZ59-SSS residues
	1	5-ring	CB/ Ala981	pi-H	3.86	−1	
	1	6-ring	CA/ Met982	pi-H	4.11	−0.6	
3G5U	1	O 17	ND2/ Asn717	H-acceptor	2.85	−1.8	QZ59-SSS upper and lower and QZ59-RRR residues
	1	6-ring	OH/ Tyr303	pi-H	3.84	−2.1	
	1	5-ring	NE2/ Gln721	pi-H	3.78	−3.8	
	2	5-ring	CD1/ Leu300	pi-H	3.74	−0.6	
3G5U	1	6-ring	6-ring/ Phe71	pi-pi	3.42	−0.0	QZ59-SSS upper and lower and QZ59-RRR residues[a]
	2	6-ring	6-ring/ Phe71	pi-pi	3.98	−0.0	
3G5U	1	C 28	6-ring/ Phe299	H-pi	3.67	−1	Verapamil binding residues
	1	5-ring	CB/ Ala338	pi-H	4.4	−0.6	
	1	6-ring	CB/ Ala338	pi-H	4.23	−0.7	
3G5U	1	6-ring	OH/ Tyr303	pi-H	3.63	−1.5	QZ59-RRR binding residues
	2	5-ring	NE2/ Gln721	pi-H	4.12	−0.6	
Human	1	6-ring	CA/ Gly346	pi-H	3.68	−0.9	Upper and Lower QZ59-SSS binding residues
Human	1	O 17	NE2/ Gln725	H-acceptor	3.2	−1.6	Upper and Lower QZ59-SSS binding and QZ59-RRR residues
	1	6-ring	CB/ Ile868	pi-H	4.46	−0.9	

(*Continued*)

ABCB1 Structure	Pose	Ligand	Atom/group in residue/	interaction	Distance (Å)	E (kcal/mol)	Binding site used
	1	6-ring	CD1/ Ile868	pi-H	4.28	−0.9	
Human	1	6-ring	CB/ Phe303	pi-H	4.03	−0.7	QZ59-SSS upper and lower and QZ59-RRR residues[a]
	1	5-ring	6-ring/ Phe303	pi-pi	3.97	−0.0	
Human	1	N 30	OG/ Ser992	H-donor	3.15	−0.7	Verapamil binding residues
	1	5-ring	CA/ Gly872	pi-H	4.37	−1.2	
Human	1	5-ring	CA/ Tyr310	pi-pi	3.54	−0.6	QZ59-RRR binding residues

[a]Conformational search was performed prior to docking and 100 docking poses were retained.

Supplementary Table 6A: Concentration-dependent influence of enzastaurin on the sensitivity of ABCG2-expressing UKF-NB-3^{ABCG2} cells to the ABCG2 substrate mitoxantrone. Cell viability was determined after a 5 day incubation period by MTT assay. Concentrations that reduced cell viability by 50% (IC_{50}) were calculated.

		Influence of enzastaurin on the mitoxantrone IC_{50}		
Enzastaurin (µM)	IC_{50}[1] mitoxantrone (ng/ml)	cell viability enzastaurin (% control)	IC_{50} mitoxantrone (ng/ml)	Fold sensitisation[2]
0.3125	59.28 ± 19.01	91.50 ± 8.35	16.51 ± 3.52[3]	3.59
0.625	66.16 ± 12.63	92.31 ± 14.51	11.23 ± 2.61[3]	5.89
1.25	54.91 ± 10.27	90.42 ± 13.62	6.17 ± 0.08[3]	8.90
2.5 µM	61.74 ± 11.42	84.89 ± 7.86	2.90 ± 0.04[3]	21.29

[1]Values are mean ± S.D. of three independent experiments.
[2]IC_{50} mitoxantrone/ IC_{50} mitoxantrone in the presence of enzastaurin.
[3]$P < 0.05$ relative to IC_{50} mitoxantrone in the absence of enzastaurin as indicated by *t*-test.

Supplementary Table 6B: (*Continued*) Influence of enzastaurin on the sensitivity of non-ABCG2-expressing UKF-NB-3 cells to the ABCG2 substrate mitoxantrone. Cell viability was determined after a 5 day incubation period by MTT assay. Concentrations that reduced cell viability by 50% (IC_{50}) were calculated.

		Influence of enzastaurin on the mitoxantrone IC_{50}		
Enzastaurin (μM)	IC_{50}[1] mitoxantrone (ng/ml)	cell viability enzastaurin (% control)	IC_{50} mitoxantrone (ng/ml)	Fold sensitisation[2]
0.3125	0.23 ± 0.05	102.17 ± 17.44	0.19 ± 0.03	1.21
0.625	0.20 ± 0.02	95.52 ± 18.65	0.18 ± 0.01	1.11
1.25	0.19 ± 0.04	81.79 ± 10.46	0.19 ± 0.03	1.00
2.5 μM	0.22 ± 0.03	67.59 ± 5.08	0.18 ± 0.05	1.22

[1]Values are mean ± S.D. of three independent experiments.
[2]IC_{50} mitoxantrone/ IC_{50} mitoxantrone in the presence of enzastaurin.

Supplementary Table 6C: (*Continued*) Influence of enzastaurin on the sensitivity of non-ABCG2-expressing UKF-NB-3 transduced with a control vector (UKF-NB-3^{iG2}) cells to the ABCG2 substrate mitoxantrone. Cell viability was determined after a 5 day incubation period by MTT assay. Concentrations that reduced cell viability by 50% (IC_{50}) were calculated.

		Influence of enzastaurin on the mitoxantrone IC_{50}		
Enzastaurin (μM)	IC_{50}[1] mitoxantrone (ng/ml)	cell viability enzastaurin (% control)	IC_{50} mitoxantrone (ng/ml)	Fold sensitisation[2]
0.3125	0.25 ± 0.06	97.81 ± 12.27	0.22 ± 0.05	1.14
0.625	0.22 ± 0.05	105.13 ± 9.84	0.23 ± 0.04	0.96
1.25	0.21 ± 0.06	86.02 ± 11.70	0.17 ± 0.02	1.24
2.5 μM	0.23 ± 0.02	63.67 ± 10.74	0.17 ± 0.06	1.35

[1]Values are mean ± S.D. of three independent experiments.
[2]IC_{50} mitoxantrone/ IC_{50} mitoxantrone in the presence of enzastaurin.
